# Dissecting the midlife crisis: disentangling social, personality and demographic determinants in social brain anatomy

**DOI:** 10.1038/s42003-021-02206-x

**Published:** 2021-06-17

**Authors:** Hannah Kiesow, Lucina Q. Uddin, Boris C. Bernhardt, Joseph Kable, Danilo Bzdok

**Affiliations:** 1 Department of Psychiatry, Psychotherapy, and Psychosomatics, RWTH Aachen University, Aachen, Germany; 2 Department of Psychology, University of Miami, Coral Gables, FL USA; 3 McConnell Brain Imaging Centre, Montreal Neurological Institute and Hospital, McGill University, Montréal, QC Canada; 4 Department of Psychology, University of Pennsylvania, Philadelphia, PA USA; 5 Department of Biomedical Engineering, McConnell Brain Imaging Centre, Montreal Neurological Institute (MNI), Faculty of Medicine, McGill University, Montréal, QC Canada; 6 Mila – Quebec Artificial Intelligence Institute, Montréal, QC Canada

**Keywords:** Computational biology and bioinformatics, Neuroscience

## Abstract

In any stage of life, humans crave connection with other people. In midlife, transitions in social networks can relate to new leadership roles at work or becoming a caregiver for aging parents. Previous neuroimaging studies have pinpointed the medial prefrontal cortex (mPFC) to undergo structural remodelling during midlife. Social behavior, personality predisposition, and demographic profile all have intimate links to the mPFC according in largely disconnected literatures. Here, we explicitly estimated their unique associations with brain structure using a fully Bayesian framework. We weighed against each other a rich collection of 40 UK Biobank traits with their interindividual variation in social brain morphology in ~10,000 middle-aged participants. Household size and daily routines showed several of the largest effects in explaining variation in social brain regions. We also revealed male-biased effects in the dorsal mPFC and amygdala for job income, and a female-biased effect in the ventral mPFC for health satisfaction.

## Abstract (web summary)

Hannah Kiesow et al. combine 40 behavioral indicators and neuroimaging data from the UK Biobank to investigate how the transitions in midlife in the domains of social, personality, and demographic determinants impact brain anatomy. Through Bayesian analyses, the authors were able to disentangle which specific traits, relative to other considered candidate traits, contributed the most to explaining differences in social brain volume.

## Introduction

Humans are inherently social organisms. As early as the first days of life, infants show signs of distress in the absence of social stimulation^1^. As humans grow older, a thirst for social embeddedness persists and may even intensify^2,3^. At midlife, roughly between the ages of 40–70^4–7^, adults have gathered decades of social experience that shape how they navigate their daily social encounters. For example, the ability to form accurate social judgments about other individuals is known to mature throughout life^8^. The continuous refinement of social skills well into adulthood may, in turn, mold other factors that impact how we navigate our social environments, such as personality disposition and demographic standing. Midlife is a special life period when many important milestones have often been reached, such as creating a family and establishing oneself in an occupation^6^.

For example, during midlife, one’s overall social network size usually starts to shrink^9,10^. When choosing with whom to spend time, middle-aged participants prefer familiar over new social interaction partners, compared with younger people^11^. Similarly, middle-aged men and women preferentially interact with close family members^12^. The consolidation of social circles towards a select core confers a feeling of social embeddedness and promotes well-being^10,13^. Overall, there is an age-related tendency to relatively disengage from social ties at the network periphery and prioritize spending time with emotionally close others^10^. As such, midlife can be viewed as a pivotal period in the lifespan when adults transition their focus from exploring new social relationships to fostering existing social connections.

The typical midlife changes in social network configuration are likely accompanied by changes in brain architecture. At this stage of life, hints from the neuroimaging literature suggest that structural alterations in frontal or prefrontal brain regions may occur as a part of normal aging^14,15^. Importantly, the loss in brain volume during healthy aging is not evenly distributed across the brain^16,17^. In a previous structural brain-imaging study in 547 participants aged 19 to 86, age-related reductions in gray matter volume were observed especially in the frontal and parietal lobes, including regions of the prefrontal cortex^18^. Indeed, the prefrontal cortex, among other frontal cortex regions, has been emphasized to show the strongest age effects in gray matter structure as people grow older, compared with the rest of the brain^5,14,15,17–22^ (but see these references reporting no such changes:^16,23^). Structural imaging research based on T1-weighted brain scanning has so far struggled to attach unambiguous meaning to findings of more or less gray matter volume in specific brain locations^24^. An increase in gray matter volume could indicate higher density of cell populations, including neurons and their substructures like cell bodies or axons. While an increase of this quantity has repeatedly been observed to be associated with enhanced cognitive performance, various counterexamples have reported reduced functional capacity. In particular, neuronal pruning processes are one candidate mechanism for how less regional volume may allow for computational efficiency gains in a specific cognitive process^24^.

Previous brain-imaging research suggests that the medial prefrontal cortex (mPFC) serves as a common computational resource for separate domains of social cognition^25^, including cognitive processes that implicate self-concept or thinking about other people’s mental states^25,26^. In parallel to how brain structure differs as a function of age, these social cognitive processes may also show characteristic changes across the lifespan. For example, a previous functional brain-imaging study investigated differences between younger and older adults in understanding the mental state of other individuals^27^. In a battery of social-cognitive tasks, the authors found older adults to consistently show deficits compared with younger adults in understanding the thoughts and actions of others, accompanied by isolated reductions in mPFC task responses. Moran and colleagues suggested specific involvement of the mPFC in mentalizing skills, and proposed that aging is associated with impairments in processing the intentions and internal states of other people^27^.

In addition to capacities implicated in social interaction, neural activity in the mPFC has also been closely linked to other key domains of everyday life, especially personality and demographics^25,28,29^. An individual’s personality is an important source of interindividual variability in approaching everyday life and reflects an individual’s disposition in their thought processes and behavior^30^. For example, higher levels of the personality trait neuroticism in early adulthood were associated with overall lower well-being later on in midlife and less positive relations with other individuals^31^. Indeed, individuals with higher levels of neuroticism were shown to have a lower quality of social relationships with others in general and romantic relationships in particular^32^. Moreover, a previous structural brain-imaging study found that higher levels of neuroticism were associated with gray matter loss in the mPFC in adults beginning around the age of 44^28^. However, the same study found that ranking high on the personality trait conscientiousness, a trait associated with positive and satisfying romantic relationships^32^, was linked to larger mPFC volumes and less gray matter decline^28^. Furthermore, another structural brain-imaging study found that extraversion, a trait associated with seeking and engaging in satisfying social interactions, was linked to increased cortical thickness in the prefrontal cortex in adults in late midlife^30^. The collective evidence suggests that personality traits may have an enduring influence on the social behavioral patterns of individuals, and are linked to correlates in brain morphology.

At the broader societal level, interindividual differences in social interaction tendencies also depend on the demographic characteristics of one’s place in society, such as economic resources, occupational prestige and education attainment^33,34^. Indeed, a brain-imaging study interrogated the relationship between gray matter volume and socioeconomic disadvantage, a composite measure of household poverty level, public assistance, education level, employment status and household income^35^. The authors reported smaller gray matter volume in the mPFC for middle-aged adults who experience more socioeconomic hardship compared with adults without precarious socioeconomic circumstances. Similarly, a previous structural brain-imaging study (*n* = 431) found that experiencing current financial hardship during midlife was also linked to smaller gray matter volume in the hippocampus and amygdala, two limbic regions with direct axonal connections to the mPFC^36^. Furthermore, Butterworth and colleagues found that middle-aged adults with financial hardship had fewer close social relationships, such as a partner, compared to middle-aged adults more financially stable. The authors speculated that experiencing financial hardship may be a source of stress on an individual, with downstream consequences of reduced brain structure due to corticosteroid exposure^36^. Correspondingly, a previous brain-imaging study (*n* = 359) linked high socioeconomic status to thicker cortical gray matter in brain regions including the prefrontal cortex, in middle-aged adults (ages 35–64) compared to middle-aged adults with a lower socioeconomic standing^37^. This effect was not observed among the younger (ages 20–34) and older (ages 65–89) age groups^37^. Chan and colleagues suggest that higher social status may be protective against age-related brain decline. These structural brain-imaging studies exploring aspects of demographics thus suggest that differences in broader sociocultural experiences may have characteristic imprints in mPFC structure in middle-aged individuals.

In these ways, earlier neuroimaging findings have highlighted the medial prefrontal cortex as a hub that bridges key factors at the individual, interpersonal, and societal level. These disparate domains are usually studied in isolated literature streams, although they may reflect common or distinct manifestations in the mPFC of the human social brain. Factors of personality traits and social behavior dispositions may have inadvertently influenced each other in previous social neuroscience studies, since these indicators were unlikely to be assessed or assessable in the same kind of quantitative analysis (e.g., refs. ^38,39^). Additionally, many such neuroscience studies have been based on small participant samples as well as sampled participants of student age^40^.

To supplement these earlier research efforts, we have tailored a probabilistic generative modeling approach to jointly study a rich set of measures from three complementary lifestyle domains, with their extent of similarity and divergence in brain volume associations. Our fully probabilistic modeling framework directly tested against each other 40 traits—tracking everyday experiences in the social, personality, and demographic domains in 10,000 UK Biobank participants in midlife. As the key advantage of this modeling tactic, we could carefully dissect the unique contribution of each examined trait, after accounting for the respective other traits, to explaining regional gray matter variation in the social brain. Given previous findings in the neuroimaging literature, we hypothesized that we will observe the most prominent effects in the medial prefrontal cortex and limbic temporal system in our brain-behavior analyses of brain structure. This expectation reflects the earlier proposition that the medial prefrontal cortex may act as a hub for different aspects of social cognition^25^, including those that relate to personality, interpersonal exchange, and societal dynamics.

## Results

By quantitatively mining the UK Biobank resource, we could juxtapose an envelope of 40 lifestyle factors in the context of social brain. Our analyses provide an alternative perspective on the question of how the measured traits are reflected in brain structure in middle age (Table 2; 40–69 years at recruitment). The examined traits offered by the UK Biobank cohort can be placed into three main domains: (i) social exchange, (ii) personality profile and (iii) demographic status (Supplementary Data 1). By integrating all of these indicators into the same analysis framework, we were able to disentangle which specific traits, relative to the other considered candidate traits, contributed most to explaining social brain volume (i.e., each trait’s marginal association or partial correlation with region variation). We estimated 36 different probabilistic models, one for each target region in the social brain atlas. Henceforth, the term ‘trait effect’ refers to the marginal posterior parameter distributions that were obtained from the estimated probabilistic models. The inferred quantities expose the magnitude, directionality, and model uncertainty in the brain association of the collective analyzed traits (cf. Methods).

### Social brain midline: overview of key findings

During midlife, parts of the prefrontal cortex were previously shown to undergo structural reorganization^5,14,15,17–22^. We paid special attention to these regions in the prefrontal cortex because they are also known to assist in realizing social cognition (e.g.,^25^), including broader social aspects that may bear relation to personality and demographics. In addition, previous research has shown that the mPFC has direct connectivity inputs to key limbic input regions, especially the amygdala and hippocampus^41–44^ from the medial-temporal system^45^. Thus, we tested the hypothesis that there would be a medial prefrontal cortex and limbic temporal dominance in age effects. Yet, in disagreement with our primary hypothesis, midline regions did not show the strongest trait effects in comparison to those of the other social brain regions. However, we did observe several noteworthy brain-trait associations in limbic and midline regions.

**Fig. 1:**
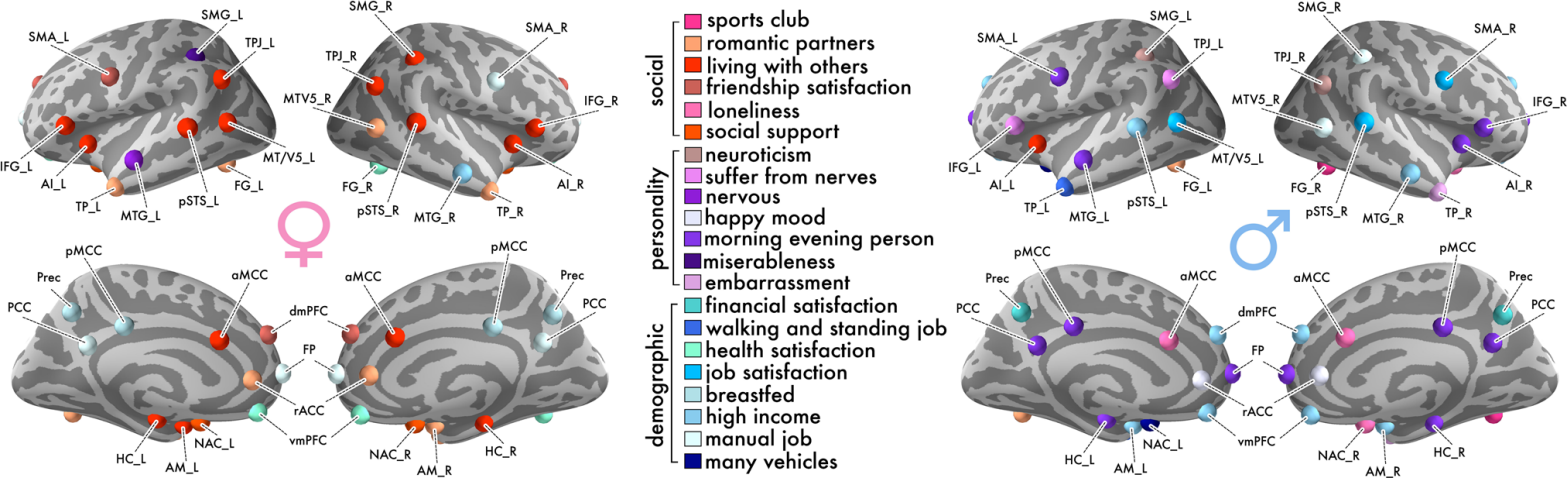
Social brain variation is preferentially explained by social traits in women, and by personality and demographic traits in men. Extending previous social neuroscience studies, the richness of the UK Biobank resource allowed uniquely isolating marginal correlations, which prevail over a wide variety of competing explanatory factors. A generative probabilistic model was estimated for each of the 36 social brain regions in our population sample of middle-aged adults. These region-by-region analyses revealed numerous dominant specific (i.e., partial) trait associations in social brain structure. Colors indicate which individual traits have the largest magnitude (i.e., strongest positive or negative association) in explaining its regional gray matter volume, relative to the other 39 out of 40 total examined traits (cf. Supplementary Data 1). Red indicates markers in the social trait category, purple indicates the personality trait category, and blue indicates the demographic trait category. Sharing one’s home with other individuals was the single most frequent trait association to show the largest magnitude in explaining social brain volume for women, in atlas regions including the AI, AM, HC, IFG TPJ and pSTS (left). The personality trait of being a morning person was the most common trait to show the strongest trait association in social brain structure for men, in atlas regions including the AI, HC, FP, IFG, PCC and pMCC (right). Dominant trait associations from the partial correlation analysis are shown in Supplementary Fig. 1 (cf. Table 3 and Supplementary Data 2 for a description of the social brain region abbreviations).

Within the mPFC and its limbic partner inputs, our study aimed to disentangle trait effects linked to social dynamics from trait effects linked to personality features and demographic status. Our results revealed that several traits related to the richness of one’s daily social encounters contributed to explaining social brain volume in the mPFC and its limbic connections (Fig. 1).

For example, sharing the home environment with other individuals emerged as a top contributor to explaining gray matter volume in the AM_L and bilateral HC (see Supplementary Data 2 for abbreviation list of the social brain regions; women: AM_L: mean of the population trait posterior distribution = 0.035, highest posterior density interval (HPDI) of the population trait posterior distribution covering 95% model uncertainty = 0.005–0.069; HC_L: posterior mean = 0.040, HPDI = 0.012–0.072; HC_R: posterior mean = 0.056, HPDI = 0.019–0.093). In parallel with these dominant trait findings on household size, markers related to interaction with close others, such as friends, showed unique trait associations in our middle-aged population cohort, relative to the other 39 competing traits. For example, feeling satisfied with one’s friendships was a top trait association in the higher associative dmPFC region (women: posterior mean = 0.039, HPDI = 0.000–0.078). Moreover, we also observed that the lifetime number of romantic partners showed a strong trait effect in the AM_R (women: posterior mean = 0.031, HPDI = 0.009–0.056).

In addition to traits characterizing aspects of one’s social lifestyle, we also found personality effects in regions of our social brain atlas (Fig. 1). Specifically, being a morning versus evening person showed the largest magnitude in explaining gray matter volume in the FP and bilateral HC, compared to the other candidate traits (men: FP: posterior mean = 0.052, 95% HPDI = 0.002–0.104; HC_L: posterior mean = 0.051, HPDI = 0.020–0.084; HC_R: posterior mean = 0.054, HPDI = 0.022–0.087).

**Fig. 2:**
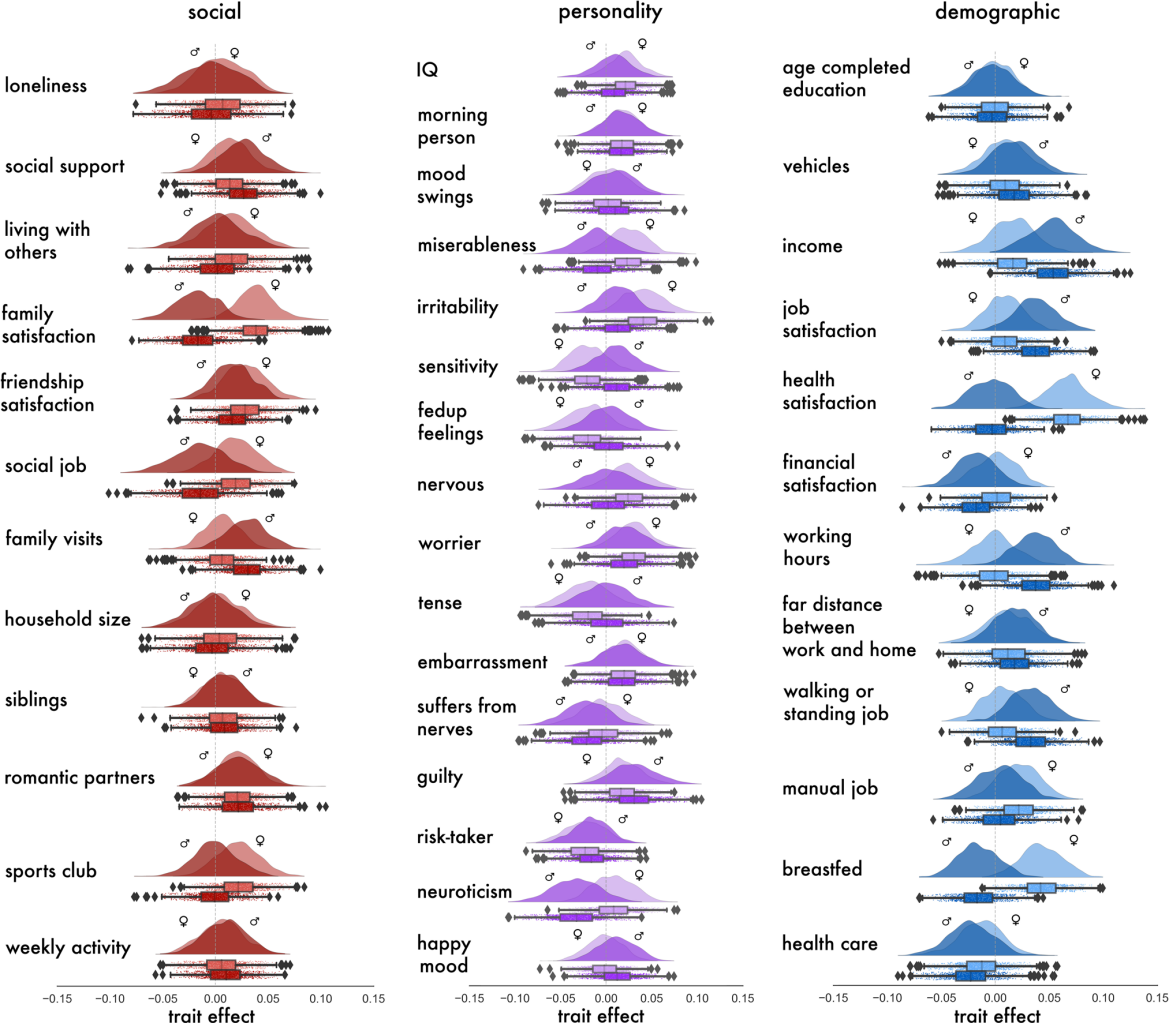
vmPFC anatomy is linked to social lifestyle markers of social, personality, and demographic traits with sex-differentiated population effects. During midlife, regions of the mPFC have been found to show an accelerated decline in gray matter structure (cf. introduction). For the sake of illustration, the *marginal* posterior population distributions from our vmPFC analysis are depicted using raincloud plots (1 of the 36 region-by-region analyses that we have conducted on the UK Biobank). We reveal to what extent vmPFC region volume is specifically explained by the 40 examined lifestyle traits at the population level (cf. Supplementary Data 1). The half violin plots show the posterior parameter distributions of the specific contributions of each single trait to vmPFC volume, not explained by the other traits, in middle-aged men and women. The boxplots and scatterplots underneath depict the probabilistic parameter guesses that together form the marginal posterior distributions. Each brain-behavior HPDI communicates three kinds of information: direction (e.g., a positive parameter value indicates a tendency for a higher brain volume in the presence of that particular trait), magnitude (e.g., a parameter mean further above or below zero indicates a bigger dependence of region volume on that particular trait), and certainty (e.g., a narrower interval indicates that the model is more sure about the estimated direction and magnitude of a particular brain-behavior effect). For middle-aged women, satisfaction with health contributed most to explaining vmPFC region volume. In contrast, for middle-aged men, earning a higher job income explained most vmPFC gray matter volume. Error bars/dispersion shows uncertainty of Bayesian posterior parameter distributions. Source data are provided in Supplementary Data 3.

Regarding traits capturing the societal level, we observed several demographic indicators to show unique trait effects in midline social brain regions and limbic inputs (Figs. 1 and 2). Our posterior parameter distributions showed that earning a high yearly income explained the biggest fraction of region volume in the vmPFC and bilateral AM, compared with the other considered traits (men: vmPFC: posterior mean = 0.053, 95% HPDI = 0.011–0.100; AM_L: posterior mean = 0.060, HPDI = 0.022–0.104; AM_R: posterior mean = 0.048, HPDI = 0.017–0.084). In the vmPFC, a similar strong trait effect was observed (Fig. 2). Health satisfaction, a trait plausibly linked to socioeconomic status, was the top contributor to vmPFC gray matter volume compared with the other examined traits (women: posterior mean = 0.067, HPDI = 0.030–0.105). In addition, working a manual, as opposed to a knowledge-based job, showed a dominant trait effect in the FP (women: posterior mean = 0.059, HPDI = 0.007–0.115). Taken together, our probabilistic evidence revealed specific trait effects that were mostly linked to social experience and demographics in social brain midline and limbic regions.

Moreover, the reiteration of our analyses based on partial region volumes of the social brain (cf. Methods) shed a different light on the dominant trait associations in social brain midline and limbic regions. The partial correlation analysis also revealed a richer variety of dominant trait effects across atlas regions, mostly in the category of demographic traits (Supplementary Fig. 1; cf. Supplementary Note for a full description of the partial correlation analysis results). For example, having an occupation that requires working more than 40 hours a week showed a dominant trait association in the vmPFC (men: posterior mean = 0.005, 95% HPDI = −0.006–0.016). However, completing full-time education, another trait indexing an aspect of demographic profile, showed dominant trait associations in the limbic AM, HC and FP (men: AM_R: posterior mean = 0.009, HPDI = −0.002–0.020; women: FP: posterior mean = 0.039, HPDI = 0.008–0.075; HC_L: posterior mean = −0.006, HPDI = −0.016–0.003). Taken together, the partial correlation analysis findings for the midline social brain regions revealed a wider assortment of dominant trait associations mostly linked to demographic profile.

### Social markers: household size is consistently linked to social brain structure

Our fully probabilistic region-by-region analyses revealed at the interpersonal level that social traits associated with social network composition and quality of interpersonal exchange showed consistently strong trait effects across social brain regions (Fig. 1). In our middle-aged population cohort, sharing the home environment with other individuals was the most common trait to explain the most region volume in about half of our 36 region analyses.

Living with others in the same household, as opposed to living alone, showed trait effects in regions of the higher associative network of our social brain atlas, including the bilateral TPJ (women: TPJ_L: posterior mean = 0.070, 95% HPDI = 0.015–0.127; TPJ_R: posterior mean = 0.059, HPDI = 0.010–0.109). Compared with the other candidate traits, sharing the home environment with other individuals also explained the largest fraction of volume variation in regions of the intermediate network, including the bilateral AI and bilateral IFG (men: AI_L: posterior mean = −0.045, HPDI = −0.107–0.011; women: AI_L: posterior mean = 0.056, HPDI = 0.012–0.101; AI_R: posterior mean = 0.058, HPDI = 0.024–0.100; aMCC: posterior mean = 0.070, HPDI = 0.021–0.120; IFG_L: posterior mean = 0.039, HPDI = −0.005–0.083; IFG_R: posterior mean = 0.065, HPDI = 0.014–0.118; SMG_R: posterior mean = 0.040, HPDI = −0.005–0.087). Furthermore, this social trait showed dominant population trait effects in regions of the visual sensory network of the social brain, including the bilateral pSTS and MT/V5_L (women: MT/V5_L: posterior mean = 0.050, HPDI = 0.004–0.097; pSTS_L: posterior mean = 0.058, HPDI = 0.010–0.110; pSTS_R: posterior mean = 0.049, HPDI = 0.000–0.096).

Additionally, social markers related to close interpersonal relationships also revealed large magnitudes in explaining gray matter volume in regions of the visual sensory, limbic, and higher associative networks. In particular, the lifetime number of romantic partners showed dominant trait effects in several visual sensory, limbic, and higher associative social brain regions including the FG, rACC, and TP (women: FG_L: posterior mean = 0.032, 95% HPDI = 0.005–0.060; MTV5_R: posterior mean = 0.054, HPDI = 0.012–0.094; TP_L: posterior mean = 0.037, HPDI = 0.010–0.067; TP_R: posterior mean = 0.033, HPDI = 0.000–0.065; rACC posterior mean = 0.057, HPDI = 0.018–0.099; men: FG_L: posterior mean = 0.048, HPDI = 0.009–0.086). This pattern of trait effects linked to social interaction with close family and friends extended to a region of the visual sensory network. Specifically, being a member of a sports club, a social trait related to regular involvement in social groups, showed the largest trait effect in the FG_R (men: posterior mean = 0.045, HPDI = 0.002–0.086).

In interindividual differences of social support, regular exchange with emotionally close others explained the most region volume in the bilateral NAC, compared with the other analyzed traits (women: NAC_L: posterior mean = 0.076, 95% HPDI = 0.018–0.133; NAC_R: posterior mean = 0.040, HPDI = −0.010–0.091). In a related social marker indexing the quality of close relationships, feelings of loneliness showed a unique trait association in several limbic and intermediate network brain regions, including the reward-related NAC (men: NAC_R: posterior mean = −0.051, HPDI = −0.128–0.020; aMCC: posterior mean = −0.051, HPDI = −0.114–0.011). Collectively, dimensions on the strength of social closeness to close family and friends were associated most with gray matter volume in a majority of social brain regions. Notably, sharing a home with other individuals was the most frequently observed dominant trait association in our participant sample.

Our reanalyses based on partial volume correlations revealed dominant trait associations similar to that of the main analysis (cf. Supplementary Fig. 1; cf. Supplementary Note for a full description of the partial correlation analysis results). For example, having a job that requires much social interaction was a most frequent trait to contribute to social brain volume in regions of the visual sensory, limbic, and intermediate brain networks (women: FG_R: posterior mean = −0.015, 95% HPDI = −0.036–0.004; IFG_L: posterior mean = −0.004, HPDI = −0.020–0.008; NAC_R: posterior mean = −0.008, HPDI = −0.025–0.007; SMA_L: posterior mean = −0.010, HPDI = −0.031–0.009). As such, our social trait findings from the partial correlation analysis here identified a similar set of dominant trait associations to that of the main analysis.

### Personality markers: top brain associations are explained by daily routine and well-being

Focusing on trait effects from the personality category, markers associated with daily routines and psychological well-being showed large magnitudes in explaining gray matter volume (Fig. 1). In general, the personality trait of being a morning versus evening person explained the largest fraction of volume variation in 11 different social brain regions, compared to the other candidate traits (Fig. 1). This biorhythm indicator showed dominant trait effects in regions of the intermediate network of our social brain atlas, including the AI_R (men: posterior mean = 0.050, 95% HPDI = 0.015–0.089), bilateral CB (men: CB_L: posterior mean = 0.069, HPDI = 0.016–0.123; CB_R: posterior mean = 0.083, HPDI = 0.028–0.140), SMA_L (men: posterior mean = 0.056, HPDI = 0.014–0.101), and IFG_R (men: posterior mean = 0.058, HPDI = 0.015–0.106). Morning versus evening chronotype also contributed to gray matter volume in regions of the higher associative network, such as the MTG_L (men: posterior mean = 0.043, HPDI = 0.006–0.081), PCC (men: posterior mean = 0.051, HPDI = 0.013–0.088), and pMCC (men: posterior mean = 0.044, HPDI = 0.012–0.075).

Additionally, our region-by-region analyses identified different personality traits related to long-term well-being to show dominant trait effects in several social brain regions belonging to the limbic, intermediate, and higher associative networks. For example, for our middle-aged participants, having a happy mood showed the largest trait effect in the limbic rACC (men: posterior mean = 0.059, 95% HPDI = 0.017–0.100). However, neuroticism explained the most variation in several regions of the intermediate and higher associative networks, including the TPJ_R, compared to the other analyzed traits (men: TPJ_R: posterior mean = −0.044, HPDI = −0.106–0.015; SMG_L: posterior mean = −0.050, HPDI = −0.113–0.009). Taken together, the personality traits associated with daily routine schedules and personal well-being emerged top traits to explain gray matter volume in a number of social brain regions.

The supplementary partial correlation analysis revealed a wider variety of personality traits to explain social brain region volume in our middle-aged population cohort (Supplementary Fig. 1; cf. Supplementary Note for a full description of the partial correlation analysis results). For example, the feeling of miserableness revealed dominant trait associations in several intermediate and higher associative social brain regions including the TP_R (men: posterior mean = 0.006, 95% HPDI = −0.007–0.020) and SMG_L (women: posterior mean = 0.014, HPDI = −0.010–0.041). Similarly, we observed the risk-taking indicator to show dominant trait associations in the limbic HC_R (men: posterior mean = −0.011, HPDI = −0.024–0.001) and intermediate SMG_R region (women: posterior mean = 0.008, HPDI = −0.011–0.029). As such, results from the partial correlation analysis revealed a larger range of personality traits to contribute to social brain gray matter volume, compared with the main analysis.

### Demographic markers: traits related to income and occupation are associated with limbic and higher associative brain regions

At the broader societal level, we observed a variety of demographic traits related to social status and occupation to explain social brain gray matter volume in our middle-aged population sample (Fig. 1). In particular, earning a high yearly wage showed dominant trait effects in several higher associative and visual sensory regions of our social brain atlas (men: MTG_R: posterior mean = 0.069, 95% HPDI = 0.027–0.111; pSTS_L: posterior mean = 0.065, HPDI = 0.016–0.113; women: MTG_R: posterior mean = 0.047, HPDI = 0.006–0.090).

Our region-by-region probabilistic results further revealed granularity in aspects related to one’s occupational environment. For example, working a manual job explained the most variation in four social brain regions of the visual sensory, intermediate, and higher associative brain networks (men: MTV5_R: posterior mean = 0.042, 95% HPDI = −0.007–0.091; SMG_R: posterior mean = 0.058, HPDI = 0.011–0.108; women: PCC: posterior mean = 0.042, HPDI = 0.009–0.074; SMA_R: posterior mean = 0.070, HPDI = 0.025–0.120). Similarly, having a job that requires walking or standing for most of the workday showed a dominant trait effect in the higher associative TP_L (men: posterior mean = 0.045, HPDI = 0.009–0.078). In the context of the work environment, feeling satisfaction with one’s occupation contributed to explaining region volume in regions of the visual sensory and intermediate networks (men: MTV5_L: posterior mean = 0.033, HPDI = −0.003–0.075; SMA_R: posterior mean = 0.042, HPDI = −0.002–0.086; pSTS_R: posterior mean = 0.059, HPDI = 0.018–0.102). Taken together, our dominant demographic trait findings at the general societal level revealed that during midlife, several aspects of one’s job environment contributed most to explaining gray matter volume variation in a variety of social brain regions.

Results from the partial correlation analysis revealed that demographic traits related to occupation were also the top contributing traits in a number of social brain regions (cf. Supplementary Fig. 1; cf. Supplementary Note for a full description of the partial correlation analysis results). For example, feeling satisfied with one’s occupation was the most frequent trait to contribute to social brain volume in several limbic, intermediate, and higher associative network brain regions (men: AI_R: posterior mean = −0.011, 95% HPDI = −0.032–0.002; IFG_R: posterior mean = 0.010, HPDI = −0.011–0.034; NAC_L: posterior mean = −0.025, HPDI = −0.052–0.000; NAC_R: posterior mean = 0.007, HPDI = −0.006–0.021; Prec: posterior mean = −0.007, HPDI = −0.023–0.008; women: pSTS_R: posterior mean = 0.010, HPDI = −0.004–0.030). Similarly, the partial correlation analysis showed that working more than 40 h a week contributed to social brain gray matter volume (men: CB_L: posterior mean = −0.007, HPDI = −0.020–0.004; MTG_R: posterior mean = 0.007, HPDI = −0.008–0.024; MT/V5_L: posterior mean = −0.010, HPDI = −0.032–0.009; vmPFC: posterior mean = 0.005, HPDI = −0.006–0.016). As such, the demographic indicator related findings from the partial correlation analysis revealed that the strongest trait associations with social brain gray matter volume were related to occupation.

In summary, our region-by-region analyses on a diverse selection of social, personality, and demographic traits together showed manifestations in social brain gray matter structure in a population cohort of adults. Compared with the social and demographic domains, traits from the personality category showed less dominant trait associations for our participant sample. Instead, markers indexing social exchange at the interpersonal and broader societal level contributed more to explaining volume variation in social brain regions during midlife.

### Participant age drives trait associations dominant in mPFC and limbic regions

**Fig. 3:**
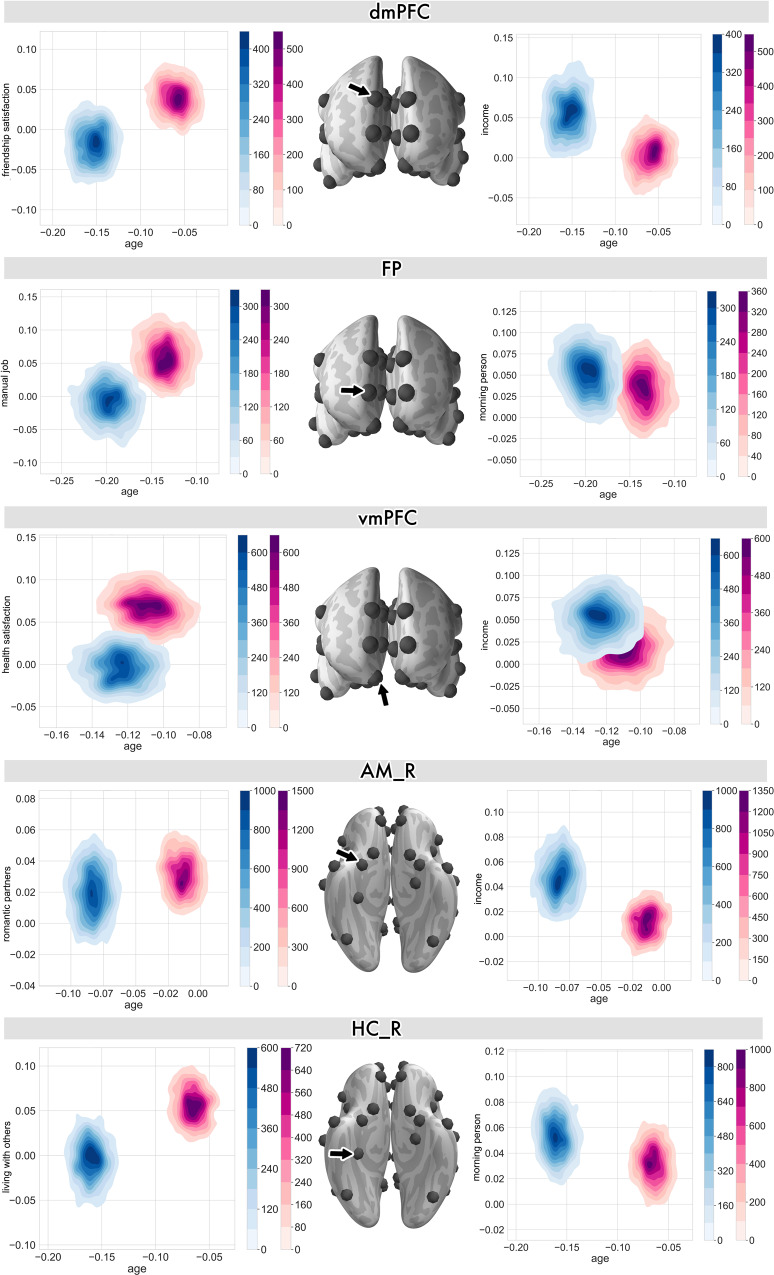
Participant age is a driving factor in how dominant lifestyle traits are linked to midline social brain regions. The co-relationship between age and a trait association in explaining region variation is quantified by the *joint* posterior parameter distribution for one particular social brain region (black arrow) for men (blue) and women (pink). This summary visualization exposes the traits with top effects in the full analysis (cf. Fig. 1; cf. Supplementary Data 2 for a description of the social brain region abbreviations). The left column shows these brain-trait associations for women and the right column shows the top trait contributions for men. Middle-aged men and women showed diverging age-trait associations in dmPFC volume in the context of social interaction quality and social status. However, in the context of two socioeconomic status measures, men and women were more similar in age-trait associations with vmPFC volume. The joint posteriors of trait associations in the FP revealed socioeconomic status as measured by job type and personality to show incongruent population parameter distributions. The limbic AM and HC regions additionally showed non-overlapping posterior distributions between middle-aged individuals for social network size and social lifestyle. Supplementary Fig. 3 showcases the age-trait associations in the midline and limbic regions from the partial correlation analysis. Error bars/dispersion shows uncertainty of Bayesian posterior parameter distributions.

Our analyses also revealed substantial contributions of age to jointly explaining gray matter variation together with other lifestyle traits (Fig. 3 and Supplementary Fig. 2; cf. Supplementary Fig. 3 for results from the partial correlation analysis). Furthermore, most of the joint age-trait effects in the medial prefrontal cortex and its interaction partners from the limbic system were manifested differently in our sample of men and women.

For example, in the dmPFC, age and high friendship satisfaction (the top trait association in the dmPFC for women) were together associated with largely divergent manifestations of dmPFC region volume for men and women across midlife (Fig. 3). Similarly, age and earning a higher income (the top trait association in the dmPFC for men) was differentially linked to dmPFC region volume for men and women. However, as a function of age, working a manual job was related to FP variation. For women, working a manual job was the top FP trait association (cf. Fig. 1). Similarly, age and the personality disposition of being a morning person showed only slightly overlapping posterior parameter distributions in the FP for men and women (Fig. 3). For the top female trait association in the vmPFC, high health satisfaction and age jointly explained region variation with largely incongruent posterior distributions for men and women. In conjunction with age, having a high yearly job income (the top male trait association in the vmPFC) showed overlapping model posteriors in explaining vmPFC volume variation for men and women.

Furthermore, we also observed age to jointly drive the strong trait effects in several limbic regions of the social brain that are known to have connections to the mPFC. For example, age and the number of lifetime romantic partners (the top female trait association in the AM_R) showed largely divergent posterior distributions for men and women. In a similar social context, age and sharing one’s home with other individuals (the top female trait association in the AM_L) showed largely incongruent posterior distributions for men and women (Supplementary Fig. 2). Moreover, high job income (the top male trait association in the bilateral amygdala) showed large posterior divergences in explaining gray matter volume for men and women (Fig. 3 and Supplementary Fig. 2). In the memory-related region of the social brain, age and sharing a home with other individuals influenced bilateral hippocampal architecture differently for men and women. In conjunction with age, morning chronotype (the top male trait association in the bilateral hippocampus) showed an opposite trend for hippocampal region volume in men and women (Fig. 3 and Supplementary Fig. 2).

Taken together, the joint age-trait effects revealed mostly divergent manifestations of gray matter volume in midline and limbic brain regions for men and women. For women, these population volume effects were more apparent in sociodemographic and social indicators, as a function of age. For men, co-relationships between age and sociodemographic or personality indicators were more prominent.

### Network-by-network summary: volume variation of specific regions is better explained by the collective traits

**Fig. 4:**
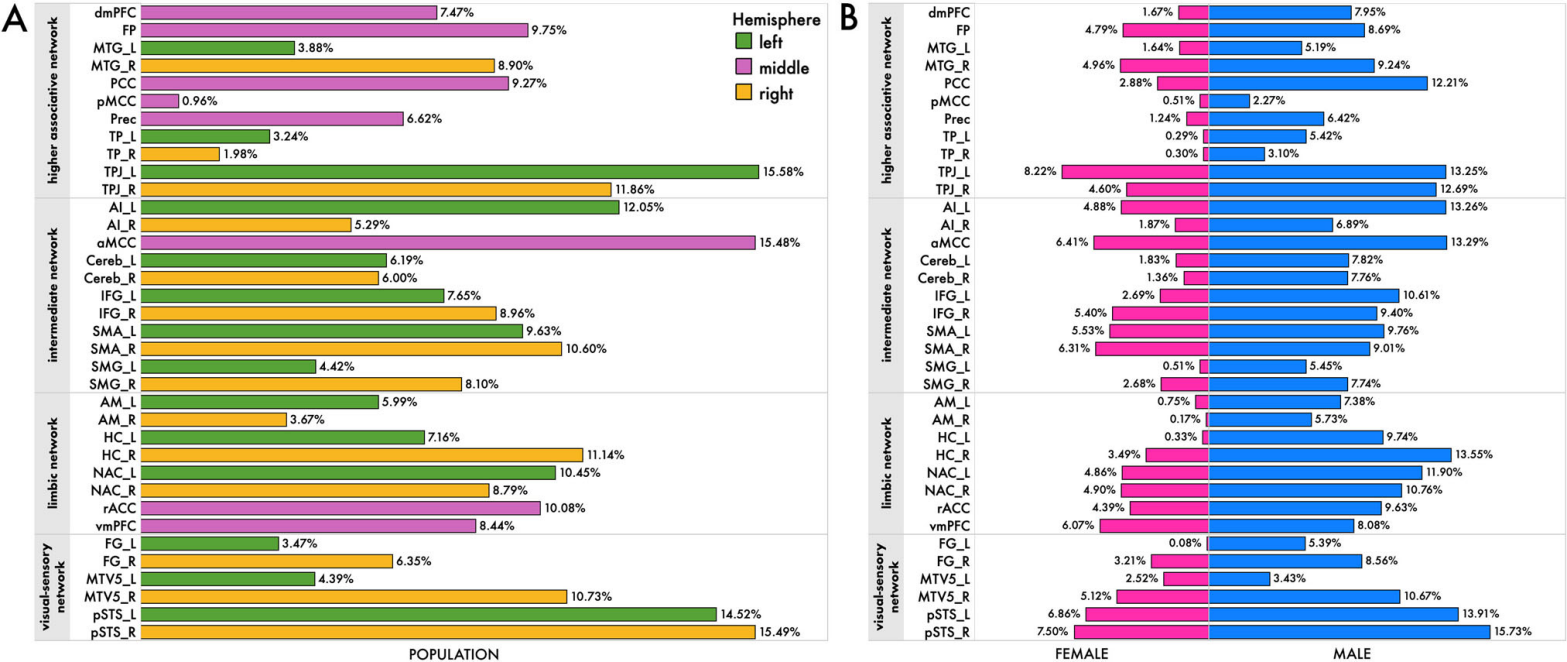
Explained variance of brain-trait associations differs across social brain networks. In each of the four networks of our social brain atlas (cf. Methods; cf. Supplementary Data 2 for a description of the social brain region abbreviations), we computed posterior predictive checks for every analysis of a given target region. These model-based simulations of replicated data were then compared to the actually observed data^47^ to compute the overall explained variance (coefficient of determination, *R*^2^) for **A** the whole population cohort and **B** men and women, separately. Posterior predictive checks thus safeguarded against several important issues related to model fit by evaluating model-simulated empirical expectations of target region volumes. Intuitively, we asked the Bayesian model: “Based on drawing examples from the previously inferred model posterior, what should the region volume in each particular participant be given his or her 40 trait indicators?”. We thus evaluated model-predicted data that could have been observed or will potentially be observed in the future. This practical check of model-based predictions of observations is a well-recognized approximation to external validation given the actual data at hand^48^. The collective population-level results suggest that in each of the four subnetworks of the social brain atlas, at least one region showed an explained variance of >10% in our middle-aged participant cohort. Source data are provided in Supplementary Data 3.

In each of the four networks of the social brain atlas (cf. Methods), we calculated posterior predictive checks for each region model. This diagnostic assessment is based on simulation of new replicated data using our previously inferred probabilistic models^46^. The simulated model outcome could then be compared to the actual observed outcomes to get a sense of our already estimated model parameter distributions. For each of the 36 examined regions from the social brain atlas, we computed the amount of explained variance from the posterior predictive checks (coefficient of determination, *R*^2^) (Fig. 4A). In this way, we interrogated which of the four networks were best explained from the 40 examined lifestyle traits for the total population sample and for men and women separately (Fig. 4B).

The total explained variance was highest for the bilateral pSTS (pSTS_R: ~16%; pSTS_L: ~15%), two regions of the visual-sensory network (Fig. 4A). In the intermediate network, aMCC and AI_L volumes showed the highest total explained variance (aMCC: ~16%; AI_L: ~12%). Instead, the TPJ_L (~16%) and TPJ_R (~12%) were the most explanatory parts of the higher associative network. In the limbic network, the HC_R (~11%) and NAC_L (~11%) showed the best *R*^2^ scores. Taken together, our results suggest that, in each of the canonical networks of the social brain atlas, at least one region was able to achieve an explained variance of >10% in our population sample.

### Sex specific trait effects are found for health satisfaction and income in midline and limbic regions

**Fig. 5:**
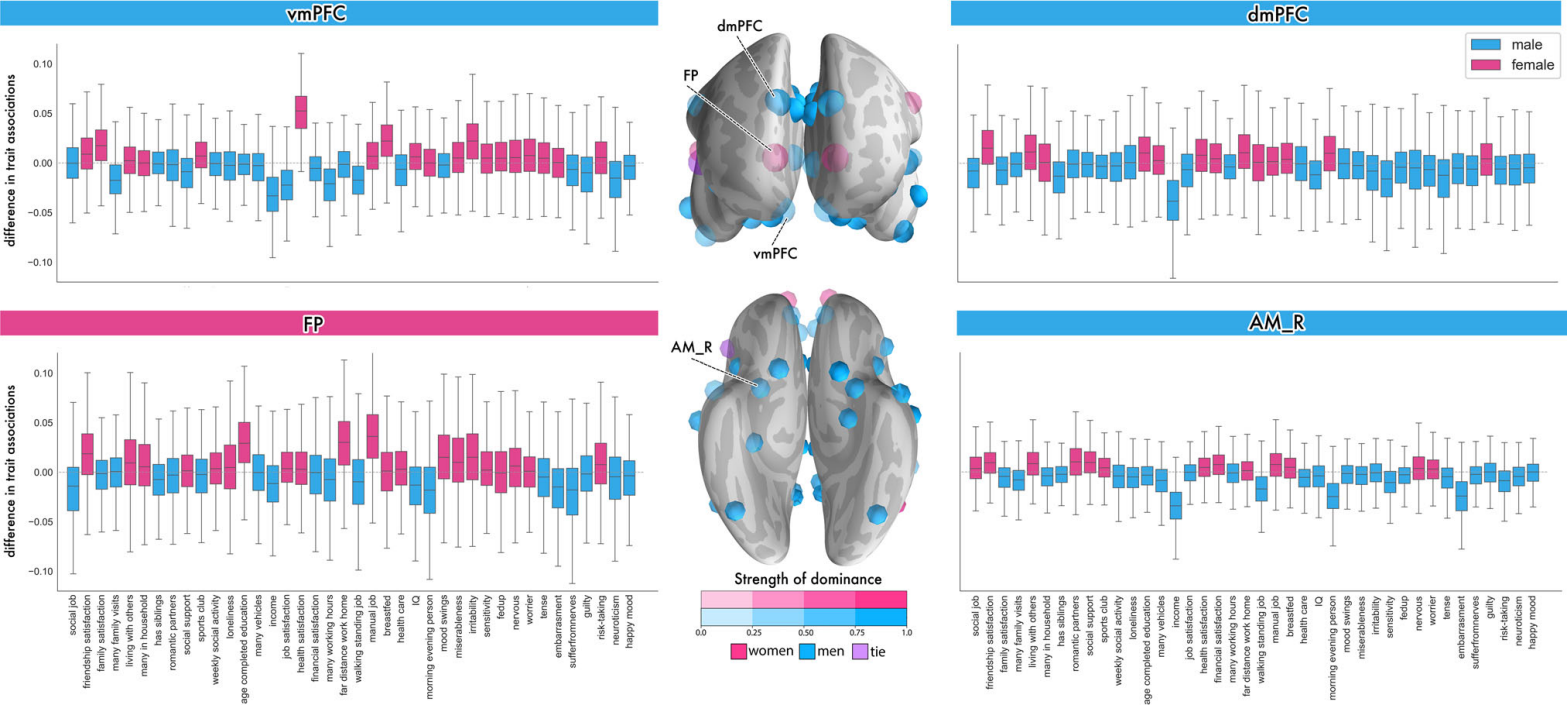
Degree of sex bias in brain-trait associations in the social brain midline. Left/Right: In each of the 40 examined traits (cf. Supplementary Data 1), boxplots show the difference contrasts between the marginal posterior population distributions of each sex (female–male). Means of posterior parameter distribution above zero indicate a relatively female-biased effect for a specific trait association (pink). For means below zero, there is a relatively male-biased effect for that specific trait (blue). Middle: To provide a summary visualization, we counted across the 40 trait associations, for each brain region, to see how many traits were biased predominantly towards males (blue) or females (pink). Purple shows an equal number of male- and female-biased trait associations. Transparency indicates the strength of the sex divergence. Overall, a male bias in volume effects becomes apparent in almost all examined medial prefrontal and limbic regions. In the dmPFC and AM_R, yearly job income showed a stronger effect in men compared to women. However, women drive the trait associations in the FP of the higher-associative social brain, especially with regards to several demographic traits, such as the age of full-time education completion and working a manual job (cf. Supplementary Fig. 5 for sex differentiation in lifestyle trait associations from the partial correlation analysis; cf. Supplementary Data 2 for a description of the social brain region abbreviations). Error bars/dispersion shows uncertainty of Bayesian posterior parameter distributions. Source data are provided in Supplementary Data 3.

Finally, we directly quantified the extent of sex differentiation in our brain-trait associations by calculating the difference between the marginal posterior distributions. We carried out the subtraction (female–male) of the model posterior parameter distributions for each trait in each social brain region analysis. The obtained difference contrasts of the  marginal posterior parameter distributions could reveal relatively more male- or more female-driven effects for a trait at hand. In the medial prefrontal regions and its limbic partners we observed more male-driven population trait effects (Fig. 5 and Supplementary Fig. 4; cf. Supplementary Fig. 5 for results from the partial correlation analysis). However, results in the FP showed more female-biased trait effects. Compared to the other midline social brain regions, the posterior distributions for the FP also showed much more uncertainty in the difference contrasts of the model posteriors.

In the vmPFC, a large female-biased trait effect became apparent. Health satisfaction contributed more to explaining volume variation in the vmPFC for women than for men. Further, in the dmPFC and bilateral amygdala regions, we observed a few specific, strong male-biased population trait effects. Notably, in the dmPFC, AM_L and AM_R, earning a higher job income showed a much larger effect for men, compared to that for women (Fig. 5 and Supplementary Fig. 4). In addition, the feeling of embarrassment and the personality trait of being a morning person showed larger trait effects in the AM_R for men compared with women. In the memory-related region of the social brain, male-specific trait effects were apparent in the bilateral hippocampus. Regular weekly contact with close individuals contributed more to explaining volume variation in the bilateral hippocampus for men than for women (Supplementary Fig. 4). However, sharing a home with other individuals showed stronger female-specific trait effects in the HC_L and HC_R. In sum, the findings from the female vs. male contrast analyses revealed degrees of sex differentiation in population associations in the limbic and higher associative regions across trait domains.

## Discussion

During midlife, many aspects of social life are subject to transition^6^. By designing a fully probabilistic modeling approach, our study interrogated social brain architecture through the prism of 40 indicators that describe the (i) social, (ii) personality, and (iii) demographic profile of ~10,000 UK Biobank participants. Many previous social neuroscience studies were limited in juxtaposing a wide breadth of lifestyle markers, partly because of the scarcity of datasets that provide deep phenotyping with a diversity of behavioral assessments. The present study confronts such a rich collection of lifestyle traits, which covers the individual, interpersonal, and broader societal level in one coherent analysis framework.

Previous neuroimaging research has reported that during this stage of life, the brain undergoes concomitant structural changes, especially in regions of the cortical midline^14,15,18^. More generally, the medial prefrontal cortex has been recognized to be a common denominator for disparate fields of neuroscience that are usually studied in isolation^25,26^, especially social interaction^27^, personality^28^, and demographics^29^. Integrating these separate results points to the medial prefrontal cortex to be a common neurocomputational resource for different processes related to daily experiences and social identity. As the hypothesis that motivated the present study, we expected to observe dominant trait effects in medial prefrontal and medial temporal limbic regions of the social brain, compared to the other atlas regions. Our collective findings did not confirm that these midline regions show the strongest volume effects for the examined target traits. In particular, our structural brain-imaging results do not fall in line with the idea that the medial prefrontal cortex acts as a singular hub for different domains of social experience. While we do find relevant brain-behavior effects in the hypothesized medial prefrontal and limbic regions, notable trait effects became apparent in several other regions of our atlas. Our middle-aged population results thus showcase in ~10,000 individuals that interindividual variation in gray matter volume in social brain atlas regions is linked to important determinants of day-to-day experience. Here, we highlight that at midlife, aspects of social support and social status may contribute most to explaining variation in the examined brain region volumes.

Key elements of daily experience include interacting with close friends and family members. Previous behavioral studies have highlighted that during midlife, adults tend to interact especially with individuals they consider to have a close, social and emotional bond with^10^. Maintaining close social bonds depends on mentalizing, or understanding the mental states of other individuals^49^. As part of the higher associative network of the social brain^50^, the dmPFC is widely acknowledged for its role in Theory-of-Mind processes^25^, which involve taking the perspective of other individuals to understand their emotions, beliefs, and motivations^51^. Here, we observed that, comparing 40 traits against each other, friendship satisfaction emerged as a dominant trait effect in the dmPFC, perhaps related to the tendency that adults prioritize maintaining close friendships during midlife.

A previous behavioral study reported that feeling unsatisfied with personal relationships is associated with the experience of loneliness^2^. Although loneliness is a subjective perception, it can have wide ranging consequences, including decreased mental and psychological well-being^52^, and even increased mortality^53^. Indeed, a cohort study on loneliness in ~900 middle-aged participants^7^ found that loneliness was linked to higher levels of stress and systemic inflammation. The authors suggest these observations to be linked to poor health outcomes, with increased risk for morbidity and mortality. In our population-level results, we observed the feeling of loneliness to be associated with gray matter volume in the NAC and aMCC. Commonly considered to be a key node of the reward circuitry, the NAC has also been found to be a neural correlate of social reward^54^. A previous structural imaging study found that individuals with a higher disposition towards social relationships displayed larger NAC volumes^54^, which may reflect the rewarding aspects of social attachment or social interaction. Our results suggest that middle-aged adults may show a sensitivity to social reward in the NAC, especially during midlife when social circles are selectively smaller and more intimate^10^.

Furthermore, Rotge and colleagues found across 46 different studies that areas of the midcingulate cortex, including the aMCC, were consistently linked to social pain, which involves the subjective feelings of loss, social disconnection, rejection or exclusion from other individuals^55^. The authors also found that being exposed to social pain for longer periods of time was linked to aMCC activity. These findings suggest a link between social brain anatomy and a role for social pain processing. These previous findings on social exclusion may relate to our finding that the trait loneliness contributed most to aMCC social brain volume. Indeed, using multimodal brain-imaging, Spreng and colleagues found that perceived isolation, or loneliness, was associated with gray matter volume, white matter integrity and functional connectivity of regions of the default mode network, including the aMCC^56^. Together, this constellation of findings on interpersonal social relations adds support to the idea that the amount of regular investments in social networks is closely linked to our structural measurements of atlas regions in the reward circuitry and intermediate network.

Consistent with the perspective that adults characteristically place their social investments on people already in their social circles during midlife, we found markers of social closeness to also relate to the AM and HC—two limbic brain regions with immediate anatomical connections to the mPFC, as evidenced in humans and monkeys^41,42,44^. Gauging 40 candidate traits, our probabilistic modeling results isolated the lifetime number of romantic partners as a top interpersonal trait to explain variation in AM volume. Consistently, previous structural brain-imaging studies have also linked the AM to variation in social group size in several age groups^57,58^. In particular, a relationship was reported between larger gray matter volume in the AM and increasing size of social networks^57^. According to these investigators’ interpretation, interindividual variation in amygdala volume is linked to differences in the complexity of one’s social life^57^. Furthermore, the lifetime number of romantic partners showed dominant trait manifestations in the limbic rACC and visual-sensory FG_L regions of our social brain atlas. These two brain regions were previously found to share functional connectivity or similar functional associations with the amygdala in the context of integrating affective and social scenes^59,60^. Together, our observed volumetric trends at population scale show that the amygdala and its close connections may have coherent links to social indices at an interpersonal level.

In a similar vein, our results from a middle-aged cohort revealed that sharing one’s home environment with others emerged as the most frequent dominant trait association across analyses in over half of the 36 target regions. Notably, these dominant trait associations were found across levels of the processing hierarchy^50^, from the pSTS and AM to HC and AI to TPJ. Previous neuroimaging studies have also shown some of these brain-trait effects based on the richness and frequency of their social interactions^57,61^. The prominence of these volumetric findings in a majority of the social brain regions may underlie middle-aged individuals’ propensity to selectively interact with close family and friends^12^. Indeed, middle-aged adults living alone often report feeling less satisfied with their personal relationships, suggesting an unmet need for belonging^2^. Together, these considerations support the perspective that during midlife, individuals typically invest resources in close relationships with friends and family.

In addition to consolidating social circles, other milestones are accomplished during midlife, such as establishing oneself in an occupation^6^. Social status is an abstract construct of individuals’ standing in society compared with others^62,63^. In our middle-aged population results, we observed that having a job that earns a high yearly income was a top contributor to gray matter region volume across visual sensory, limbic and higher associative atlas regions including the pSTS, AM, MTG, dmPFC, and vmPFC, compared with the other examined traits. As a marker of socioeconomic affluence, the amount of yearly income a person earns aids in tracking one’s own place in the hierarchical layers of society as well as the social status of other individuals^64,65^. A previous functional MRI study explored the neural processing of social hierarchies and found that participants generally tend to focus more on superior versus inferior individuals^63^. In an unstable social hierarchy, brain regions of the occipital and parietal cortex extending into the pSTS, AM and mPFC were recruited when a participant viewed a superior individual^63^. The authors interpret their findings in the pSTS, AM and mPFC in the context of emotional processing and impression formation of other people’s behavior. Hence, our findings on income as a dominant trait manifestation in several social brain regions are consistent with the possibility that social status may have volume adaptations in the frontal and temporal cortices at different levels of the processing hierarchy during midlife.

At the individual level, being more of a morning person is associated with behavioral tendencies that promote prosociality, social connection and cooperation, as well as personality traits of conscientiousness and agreeableness^66,67^. Conscientiousness has been linked to career success and overall physical and psychological well-being across the lifespan^68,69^. Compared against the other examined traits, being a morning versus evening person here showed a dominant trait association in the FP. Located in the mPFC, the FP is a region that is not only closely linked to perspective-taking capacity^70^, but also future- and goal-oriented thinking^71^. In line with our social brain-trait associations, a previous voxel-based morphometry study found morning chronotype to be associated with gray matter volume in several brain regions, including the AI, IFG, and mPFC^66^. Thus, having an early-riser biorhythm may perhaps provide far-reaching benefits during midlife, such as achieving certain career milestones.

Relatedly, our population-level results revealed that the early-bird chronotype trait from the UK Biobank also emerged as a dominant trait association in different atlas regions of the frontal, parietal and temporal cortex, including the AI, HC, IFG, MTG, PCC and pMCC. Several of these social brain regions have previously exhibited functional coactivation in meta-analytic connectivity modeling or resting state functional connectivity, such as the IFG and PCC^50^. The HC serves critical roles in memory processes, and has been thought to be involved in social monitoring, such as processes related to adhering to social norms^72^. Indeed, previous research has shown that being a morning, rather than evening, person is related to better adherence to social norms, self-control, cooperation, respecting authority, and the social desire to give off a positive impression^73^. Additionally, being more of a morning person may sometimes require overriding one’s natural sleep-wake cycle to conform to prevailing societal norms, even if one may be more of an evening person^73^. Conversely, the night-owl chronotype, as captured in the UK Biobank, has been linked to measures related to risk-taking, creativity, and resistance to acting in conventional manners^73^. The conjunction of these previous behavioral findings and our present population-level results support several parts of our social brain atlas to relate to morning versus evening orientation.

As a caveat to conclusions from our study, we used an atlas of the social brain that was derived from functional MRI data as a basis for structural MRI analyses. In a review, Suarez and colleagues describe how functional and structural MRI data do not share all properties from a signal processing perspective and expose certain differences in global organization, despite much overlap^74^. For example, the authors emphasize that, although the structural connectivity and functional connectivity of brain regions are correlated, this correspondence is imperfect. A degree of misalignment becomes especially evident in cases where brain regions are functionally connected, but not structurally connected^74^. As another example, from a mesoscopic perspective, resting-state functional connectivity networks are characterized mostly by spatially distributed systems related to cognition and perception. However, these functional networks may not be identified in brain structure, perhaps because the functional networks do not share anatomical connections^74^.

Despite these considerations on the discordance between functional and structural MRI data, we sought to link indices of personality, social interaction and demographics to gray matter brain structure. One key advantage of investigating brain structure, as opposed to investigating neural activity, is that structural measurements capture information about stable states of the individual, such as personality, that are less affected by factors such as time of day^66^. Furthermore, the regions of our social brain atlas derived using fMRI^50^ have consistently been associated with social and affective processes in brain structure in the neuroimaging literature (e.g.,^57,61,70^). In our previous work, this atlas has also served as a starting point to identify links between brain structure and social cognitive processes (e.g.,^58,75,76^). More broadly, neuroimaging studies using both functional and structural MRI data are mostly correlational in nature, by showing associative relationships between the brain and a behavioral trait, and they are typically impotent in establishing causal relationships^24^. Hence, future studies should use different techniques for intervention on the brain  such as TMS to supplement these efforts and make steps towards causal relationships between behavior and the brain^24^.

We also acknowledge that manual quality control is a challenge, and has been argued to become infeasible at the scale of several thousand participants^77,78^. Due to these constraints to manual quality control, our study relied on the automated expert pipelines for quality control and assurance from FMRIB Oxford^77,78^. This widely trusted data pre-processing workflow was specifically designed for the UK Biobank based on the first 10,000 participant data release, which is the same 10,000 participant data release on which the present study focused. However, we acknowledge that some sources of inaccuracy may have played a role in our investigation, which is challenging to exhaustively exclude given our large sample of participants.

As another limitation to the present study, the UK Biobank is a prospective epidemiological study. This initiative collected a vast portfolio of behavioral and demographic assessments, medical and cognitive measures, and biological samples. However, UK Biobank traits do not necessarily concur with measurements of classical psychological constructs that are traditionally studied in behavioral research on personality or demographics. Furthermore, it has been explicitly acknowledged that a complete picture of demographics, for example, is difficult to obtain^79^. Additionally, the usage of a limited scope of indicators may obscure a comprehensive understanding of how different experiences in an environment contribute to behavior or brain structure^33^. In this sense, we encourage the readers to interpret our results with appropriate caution. Nevertheless, the UK Biobank does provide widely-used measures of demographic standing, including household income, education attainment and occupation^79^.

As a final limitation, we admit that our approach is impotent to isolating causal relationships or ground-truth directionality in the examined effects (cf. above). However, our study illuminates behavioral factors that co-occur with brain volume manifestations in middle-aged men and women. On the one hand, our quantitative findings can annotate how the decades-long accumulation of social skills and experience in societal roles may resonate in inter-individual variation in morphological measures of the circuitry implicated in navigating social environments. As an alternative interpretation, on the other hand, distinct individual, interpersonal and societal dimensions feed into life choices and life experience well into midlife. Our population-level insights provide a glimpse into how different lifestyles may be reflected in long-term effects in social brain circuitry.

By elevating a Bayesian modeling framework to population scale, we simultaneously put to the test 40 lifestyle traits with their correspondences in the social brain. This analytical strategy allowed pinpointing some of the leading sources of population variation with imprints in brain anatomy. In offering neurobiological evidence in ~10,000 middle-aged individuals, we began to shed light on potential intersections between three disparate fields of neuroscientific inquiry: social interaction, personality disposition, and demographic constitution.

## Methods

### Population data resource

The UK Biobank initiative (UKBB) is a prospective epidemiology resource that contains a vast portfolio of behavioral and demographic assessments, medical and cognitive measures, as well as biological samples from a large cohort. ~500,000 participants were recruited across the United Kingdom^80^. This openly accessible population dataset aims to provide multimodal brain-imaging for ~100,000 individuals to be completed in 2022^78^. The present study focused on 9,939 participants who provided T1-weighted structural brain magnetic resonance imaging (MRI), comprising 48% males and 52% females. These individuals were all in middle-age, ages 40 to 69 years at the time of recruitment (mean = 55 years, SD = 7.5 years). All participants were uniformly assessed and brain-scanned at the same scanning facility (i.e., Cheadle). To ensure comparability and reproducibility with other and future UKBB studies, we relied on the data preprocessing pipelines from FMRIB Oxford^77,78^. The present analyses were conducted under UKBB application number 23827. All participants provided informed consent to participate (http://biobank.ctsu.ox.ac.uk/crystal/field.cgi?id=200).

Our present study co-analyzed a set of 40 behavioral indicators (Supplementary Data 1) provided by the UKBB resource (Table 1). The 40 summary measures belonged to three different domains: (i) social (12 items), (ii) personality (15 items), and (iii) demographic (13 items). All UKBB participants were administered questions for the particular trait measures (see here for further details: https://www.ukbiobank.ac.uk/). For example, to obtain a measure of the risk-taking trait, participants were asked “Would you describe yourself as someone who takes risks?”. According to the responses given by the UKBB participants for each interview question, participants were split into two evenly sized groups that reflect the presence or absence of the particular trait: (a) not a risk-taker and (b) risk-taker. To achieve direct comparability of the equal variable encoding in the rich collection of lifestyle indices, all target items were represented with two-choice encoding^58^.

**Table 1:** UK Biobank demographic information.

	Percent	Mean	SD	Range
Age		55	7.5	40–70
Sex				
Female	52.4			
Male	47.6			
Ethnic background				
British	27.7			
Irish	22.6			
Any other white background	2.3			
Others	3.3			
Household income (£)				
31,000–51,999	27.7			
52,000–100,000	22.6			
18,000–309,999	21.7			
<18,000	12.4			
>100,000	5.4			
Age completed (school) education		17	2.3	5–35
Body Mass Index (BMI)		26.7	4.3	16.1–63.6
Fluid intelligence score		6.65	2.04	1.0–13.0

**Table 2:** **List of the 40 examined traits available in the UK Biobank**. Each lifestyle indicator of interest from UK Biobank participants is shown alongside its field identification number. Each indicator was analyzed in two groups according to sex. All indicators were divided into one of three categories, defined by traits related to regular social interaction (social domain), traits related to personality (personality domain), and traits related to demographic standing and environment (demographic domain).

UKBB-ID	Domain	Trait	Men	Women
22617	Social	Social job (0 = less social job, 1 = more social job)	0.06 (±0.24 SD)	0.16 (±0.36 SD)
4570	Social	Friendship satisfaction (0 = low satisfaction, 1 = high satisfaction)	0.21 (±0.41 SD)	0.24 (±0.43 SD)
4559	Social	Family satisfaction (0 = low satisfaction, 1 = high satisfaction)	0.24 (±0.43 SD)	0.23 (±0.42 SD)
1031	Social	Family visits (0 = low number of visits, 1 = high number of visits)	0.34 (±0.48 SD)	0.46 (±0.50 SD)
709	Social	Living with others (0 = living alone, 1 = living with other individuals)	0.85 (±0.35 SD)	0.83 (±0.37 SD)
709	Social	Household size (0 = living with 3 or fewer housemates, 1 = living with 4+ housemates)	0.22 (±0.41 SD)	0.20 (±0.40 SD)
5057	Social	Siblings (0 = only child, 1 = has siblings)	0.87 (±0.33 SD)	0.88 (±0.32 SD)
2149	Social	Romantic partners (0 = one romantic partner, 1 = more than one romantic partners)	0.78 (±0.41 SD)	0.75 (±0.44 SD)
2110	Social	Social support (0 = low social support, 1 = high social support)	0.54 (±0.50 SD)	0.55 (±0.50 SD)
6160	Social	Sports club (0 = not in a sports club, 1 = sports club member)	0.35 (±0.48 SD)	0.36 (±0.48 SD)
6160	Social	Weekly social activity (0 = no weekly social activity, 1 = weekly social activity)	0.72 (±0.45 SD)	0.73 (±0.44 SD)
2020	Social	Loneliness (0 = not lonely, 1 = lonely)	0.12 (±0.32 SD)	0.18 (±0.38 SD)
1180	Personality	Morning/evening person (0 = evening person, 1 = morning person)	0.64 (±0.48 SD)	0.64 (±0.48 SD)
1920	Personality	Mood swings (0 = no mood swings, 1 = mood swings)	0.37 (±0.48 SD)	0.45 (±0.50 SD)
1930	Personality	Miserableness (0 = not miserable, 1 = miserable)	0.33 (±0.47 SD)	0.49 (±0.50 SD)
1940	Personality	Irritability (0 = not irritable, 1 = irritable)	0.29 (±0.45 SD)	0.25 (±0.44 SD)
1950	Personality	Sensitivity (0 = is not sensitive, 1 = sensitive)	0.44 (±0.50 SD)	0.59 (±0.49 SD)
1960	Personality	Fed-up feelings (0 = does not have fed-up feelings, 1 = has fed-up feelings)	0.32 (±0.47 SD)	0.39 (±0.49 SD)
1970	Personality	Nervous (0 = not a nervous person, 1 = nervous)	0.17 (±0.38 SD)	0.22 (±0.42 SD)
1980	Personality	Worrier (0 = not a worrier, 1 = worrier)	0.44 (±0.50 SD)	0.60 (±0.49 SD)
1990	Personality	Tense (0 = not a tense person, 1 = tense)	0.12 (±0.33 SD)	0.17 (±0.37 SD)
2000	Personality	Embarrassment (0 = does not worry too long after embarrassment, 1 = worries after embarrassment)	0.40 (±0.49 SD)	0.54 (±0.50 SD)
2010	Personality	Suffers from nerves (0 = does not suffer from nerves, 1 = suffers from nerves)	0.19 (±0.39 SD)	0.17 (±0.37 SD)
2030	Personality	Guilty (0 = is not a guilty person, 1 = guilty)	0.21 (±0.41 SD)	0.35 (±0.48 SD)
2040	Personality	Risk-taking (0 = not a risk-taker, 1 = risk-taker)	0.35 (±0.48 SD)	0.20 (±0.40 SD)
20127	Personality	Neuroticism (0 = low neuroticism, 1 = high neuroticism)	0.27 (±0.44 SD)	0.37 (±0.48 SD)
4526	Personality	Happy mood (0 = unhappy, 1 = happy)	0.81 (±0.39 SD)	0.81 (±0.39 SD)
845	Demographic	Age completed education (0 = younger age, 1 = older age)	0.22 (±0.42 SD)	0.27 (±0.44 SD)
728	Demographic	Vehicles (0 = few vehicles, 1 = many vehicles)	0.61 (±0.49 SD)	0.56 (±0.49 SD)
738	Demographic	Income (0 = low income, 1 = high income)	0.32 (±0.46 SD)	0.25 (±0.43 SD)
4537	Demographic	Job satisfaction (0 = low satisfaction, 1 = high satisfaction)	0.86 (±0.35 SD)	0.84 (±0.36 SD)
4548	Demographic	Health satisfaction (0 = low satisfaction, 1 = high satisfaction)	0.79 (±0.41 SD)	0.81 (±0.40 SD)
4581	Demographic	Financial satisfaction (0 = low satisfaction, 1 = high satisfaction)	0.80 (±0.40 SD)	0.82 (±0.38 SD)
767	Demographic	Working Hours (0 = 40 hour work week, 1 = 40+ hour work week)	0.26 (±0.44 SD)	0.11 (±0.31 SD)
796	Demographic	Distance between work and home (0 = close distance, 1 = far distance)	0.23 (±0.42 SD)	0.14 (±0.34 SD)
806	Demographic	Walking or standing job (0 = job involves mostly sitting, 1 = job involves mainly walking or standing)	0.73 (±0.44 SD)	0.73 (±0.45 SD)
816	Demographic	Manual job (0 = job does not involve heavy manual or physical work, 1 = job involves heavy or manual work)	0.48 (±0.50 SD)	0.49 (±0.50 SD)
1677	Demographic	Breastfed as Infant (0 = no, 1 = yes)	0.57 (±0.50 SD)	0.58 (±0.49 SD)
4674	Demographic	Health care (0 = public health care, 1 = private health care)	0.27 (±0.44 SD)	0.26 (±0.44 SD)
20016	Demographic	IQ (0 = low IQ, 1 = high IQ)	0.20 (±0.40 SD)	0.18 (±0.38 SD)

**Table 3:** Social brain atlas regions and their MNI coordinates. Social brain regions and their respective functional network^51^.

Social brain region	Abbreviation	MNI coordinates *x* *y* *z*	Network
Left anterior insula	AI_L	−34 19 0	Intermediate
Right anterior insula	AI_R	38 18 −3	Intermediate
Left amygdala	AM_L	−21 −4 −18	Limbic
Right amygdala	AM_R	23 −3 −18	Limbic
Anterior mid-cingulate cortex	aMCC	1 25 30	Intermediate
Left cerebellum	CB_L	−21 −66 −35	Intermediate
Right cerebellum	CB_R	28 −70 −30	Intermediate
Dorsomedial prefrontal cortex	dmPFC	−4 53 31	Higher associative
Left fusiform gyrus	FG_L	−42 −62 −16	Visual-sensory
Right fusiform gyrus	FG_R	43 −57 −19	Visual-sensory
Medial frontal pole	FP	1 58 10	Higher associative
Left hippocampus	HC_L	−24 −18 −17	Limbic
Right hippocampus	HC_R	25 −19 −15	Limbic
Left inferior frontal gyrus	IFG_L	−45 27 −3	Intermediate
Right inferior frontal gyrus	IFG_R	48 24 2	Intermediate
Left middle temporal gyrus	MTG_L	−56 −14 −13	Higher associative
Right middle temporal gyrus	MTG_R	56 −10 −17	Higher associative
Left middle temporal V5 area	MT/V5_L	−50 −66 5	Visual-sensory
Right middle temporal V5 area	MT/V5_R	50 −66 6	Visual-sensory
Left nucleus accumbens	NAC_L	−13 11 −8	Limbic
Right nucleus accumbens	NAC_R	11 10 −7	Limbic
Posterior cingulate cortex	PCC	−1 −54 23	Higher associative
Posterior mid-cingulate cortex	pMCC	−3 −29 32	Higher associative
Precuneus	Prec	−1 −59 41	Higher associative
Left posterior superior temporal sulcus	pSTS_L	−56 −39 2	Visual-sensory
Right posterior superior temporal sulcus	pSTS_R	54 −39 0	Visual-sensory
Rostral anterior cingulate cortex	rACC	−3 41 4	Limbic
Left supplementary motor area	SMA_L	−41 6 45	Intermediate
Right supplementary motor area	SMA_R	48 6 35	Intermediate
Left supramarginal gyrus	SMG_L	−41 −41 42	Intermediate
Right supramarginal gyrus	SMG_R	54 −30 38	Intermediate
Left temporal pole	TP_L	−48 8 −36	Higher associative
Right temporal pole	TP_R	53 7 -26	Higher associative
Left temporo-parietal junction	TPJ_L	−49 −61 27	Higher associative
Right temporo-parietal junction	TPJ_R	54 −55 20	Higher associative
Ventromedial prefrontal cortex	vmPFC	2 45 −15	Limbic

### Brain-imaging preprocessing procedures

MRI measurements were acquired with a 3 T Siemens Skyra scanner at the same dedicated recruitment center (i.e., Cheadle), with the same acquisition protocols and same standard Siemens 32-channel radiofrequency receiver head coils. To protect the anonymity of the study participants, brain scans were defaced and any sensitive information from the header was removed. Automated processing and quality control pipelines were deployed^77^. To improve homogeneity of the imaging data, noise was removed by means of 190 sensitivity features. This approach allowed the reliable identification and exclusion of problematic brain scans, such as scans with excessive head motion.

High-resolution T1-weighted images of brain anatomy were acquired using a 3D MPRAGE T1-weighted sequence at 1 mm isotropic resolution. Preprocessing included gradient distortion correction, field of view reduction using the Brain Extraction Tool^81^ and FLIRT^82,83^, as well as non-linear registration to MNI152 standard space at 1 mm resolution using FNIRT^84^. To avoid unnecessary interpolation, all image transformations were estimated, combined, and applied by a single interpolation step. Tissue-type segmentation into cerebrospinal fluid, gray matter, and white matter was applied using FAST (FMRIB’s Automated Segmentation Tool^85^) to generate full bias-field-corrected images. In turn, SIENAX^86^ was used to derive volumetric measures normalized for head sizes. The ensuing adjusted volume measurements represented the amount of gray matter corrected for individual brain sizes.

### Social brain atlas definition

Our study benefited from a recently available atlas of the social brain^50^, which provides a current best estimate of social brain topography in humans. This atlas resulted from quantitatively synthesizing ~4000 experimental functional MRI studies, involving thousands of participants^50^. Thirty-six convergence locations of interest (Table 3) were derived in a data-led fashion that were consistently involved in a wide assortment of social and affective tasks (see Supplementary Data 2 for stereotaxic MNI coordinates for each of the social brain atlas regions).

The 36 data-derived social brain regions are connectionally and functionally segregated into four major brain networks (cf. Supplementary Data 2): (i) a visual-sensory network (fusiform gyrus, posterior superior temporal sulcus, MT/V5), (ii) a limbic network (amygdala, ventromedial prefrontal cortex, rostral anterior cingulate cortex, hippocampus, nucleus accumbens), (iii) an intermediate network (inferior frontal gyrus, anterior insula, anterior mid-cingulate cortex, cerebellum, supplementary motor area, supramarginal gyrus), and (iv) a higher-associative network (dorsomedial prefrontal cortex, frontal pole, posterior mid-cingulate cortex, posterior cingulate cortex, precuneus, temporo-parietal junction, middle-temporal gyrus, temporal pole).

The topographical specificity of the present quantitative analyses was thus enhanced by guiding brain volume extraction of the 36 known regions of interest. Neurobiologically interpretable measures of gray matter volume were thus extracted in the ~10,000 participants. This was achieved by summarizing the whole-brain anatomical maps guided by the topographical compartments of the social brain. In particular, we applied a smoothing filter of 5 mm FWHM to the participants’ structural brain maps to homogenize local neuroanatomical differences^58,76^.

Next, gray matter volume was extracted in spheres of 5 mm diameter around the consensus location from the atlas, averaging the MRI signal across the voxels belonging to a given target region. We would like to note that using a smaller sphere diameter of 2.5 mm or a bigger one of 7.5 mm yielded virtually identical results, which led to the same conclusions. This way of engineering morphological brain features yielded 36 volume brain variables per participant, that is, as many as the total number of social brain regions. Each of the 36 brain volume variables was subsequently *z*-scored across participants by centering to zero mean and unit-variance scaling to one. These commonly employed estimates of population brain volume variability^78,87^ in social brain anatomy served as the basis for all subsequent analysis steps.

All of the regions of interest used in this study are available online for transparency and reuse at the open-data sharing platform NeuroVault (http://neurovault.org/collections/2462/).

### Probabilistic multiple regression of region variation on lifestyle traits

To explicitly interrogate the unique contribution of the 40 lifestyle traits to explaining variation in a given social brain region, we implemented a generative probabilistic multiple regression approach^58,88,89^. By adopting this Bayesian modeling framework, we could directly learn from data the specific relevance of traits spanning three different domains taken from the UK Biobank (cf. Supplementary Data 1): social behavior, personality, and demographics. In this way, we were also able to estimate the Bayesian posterior uncertainty intervals of trait effects, rather than restricting attention to rigid categorical differences. Before implementation of the region-specific probabilistic analyses, we performed a de-confounding procedure on all 36 target region volumes to remove variation due to head size and body mass index. This data cleaning step was performed in Python using nilearn (http://nilearn.github.io/, version 0.6.2). The probability models were specified as follows. For the *j*th social brain region (*j* = 1, …, 36):

1 $${y}_{j}=\beta {({\rm{j}})}_{0[{\rm{g}}]}+{x}_{1}* \beta {({\rm{j}})}_{1[{\rm{g}}]}+\ldots +{x}_{40}* \beta {({\rm{j}})}_{40[{\rm{g}}]}+{\rm{cov}}\_{\rm{age}}* \beta \_{\rm{cov}}({\rm{j}})+{\in }_{j},$$

where *y*_*j*_ is the volume of the *j*th atlas region; *x*_*i*_ is a considered social lifestyle trait and *β*(*j*)_*i*_ is the corresponding coefficient (*i* = 1,…, 40). The priors endow the model parameters *β*(*j*)_*i*_ with a Normal(0, 1) distribution for the location component and with a HalfCauchy(1) distribution for the dispersion component, while $\epsilon$_j_ is the model error which is assumed to be normally distributed with a variance component defined by HalfCauchy(5) distribution. Preliminary sensitivity analysis confirmed that small changes to our choices of prior led to virtually the same results, which was expected given the sample size of our UK Biobank cohort^89^.

Approximate posterior inference was achieved by Markov Chain Monte Carlo (MCMC) using PyMC3 in Python (https://github.com/pymc-devs/pymc3, version 3.7), which sampled in a random walk towards the target posterior distribution. In 5000 draws, the approximate parameter distributions were improved across MCMC steps in the sense of converging to the target distribution. For the partial correlation analysis (cf. below), we carried out the random walk using a number of 10,000 draws to ensure a more stable estimate of the parameter space. At each step of the MCMC chain, the entire set of parameter values were estimated to be jointly credible given the population data. A range of possible explanations for the data or parameter configurations for the relation between the social lifestyle traits and social brain volume were browsed through by obtaining multiple plausible settings of model parameters that could have led to the observed data. We searched through possible configurations of parameters as an efficient way of exploring the important part of the parameter posterior space. In particular, we dropped the first 4000 samples from the chain because (1) the chain had probably not yet fully reached stationarity and (2) this step reduced dependence on the starting parameter values. Proper convergence was assessed by ensuring the R-hat metrics stayed below 1.02^47^.

In particular, for the purpose of posterior predictive checks, in each brain-behavior analysis 500 candidate models were spawned. Each instance in that collection of possible modeling solutions embodied a different realization of the collective model parameters. In an empirical approach, the predictions generated from each of the 500 distinct possible models were then examined for plausibility against the real data at hand. We thus evaluated model-predicted data that could have been observed or will potentially be observed in the future. Any quantitative model predicts well in some situations but not in others. The used framework allowed quantifying the limits of our Bayesian models based on the cases in which they succeeded or failed.

### Reanalysis after partial correlation analysis among social brain regions

In a subanalysis of our main analysis approach (cf. previous paragraph), we introduced a preceding partial correlation step to separate out the unique portion of variation in the 36 region volumes of the social brain atlas using a linear de-correlation procedure. For one specific region among the 36 total regions, this initial processing step partialed out the volume variation shared with any of the other respective 35 social brain regions, before carrying out the actual probabilistic Bayesian analysis of interest (cf. previous paragraph).

Each of the new de-correlated residual region volumes $\epsilon$_j_ for the social brain atlas was obtained by regressing that region’s (*z*-scored) measures against the (*z*-scored) measures from all remaining regions:

2 $$	{c}y_{1}=y_{2}\,*\, {\rm{beta}}\_{\rm{a}}+{\rm{y}}_{3}\,*\, {\rm{beta}}\_{\rm{b}}+\ldots +y_{36}\,*\, {\rm{beta}}\_z+{\in }_{1}\\ 	 y_{2}=y_{1}\,*\, {\rm{beta}}\_{\rm{a}}+{\rm{y}}_{3}\,*\, {\rm{beta}}\_{\rm{b}}+\ldots +y_{36}\,*\, {\rm{beta}}\_z+{\in }_{2}\\ 	 \, \ldots \\ 	 y_{36}=y_{1}\,\ast\, {\rm{beta}}\_{\rm{a}}+{\rm{y}}_{2}\,*\, {\rm{beta}}\_{\rm{b}}+\ldots +y_{35}\,*\, {\rm{beta}}\_z+{\in }_{36}$$

where the residuals $\epsilon$_j_ represent the orthogonalized variance component of region *j* across the UK Biobank participants that could not be linearly explained by any of the 35 remaining brain region volumes. Note that the estimated values of the beta parameters were not of scientific interest in this data preprocessing step. The partialed region volumes $\epsilon$_j_ served as the basis for the otherwise identical brain-trait analyses.

In other words, the partial correlation subanalysis goes one step further and accounts for the unique variance of each of the traits in addition to accounting for the companion effects from the other social brain regions. Hence, any linear variation shared with other brain regions was removed for a particular brain region, such that the unique contribution of each trait is shown for that one specific social brain region only. In this way, we were able to provide a complementary perspective on how the 40 candidate traits are coherently associated with volume variation in the social brain in our population cohort. As such, in this variant of our original analysis, we revisited the interrogated social brain-trait associations while disregarding volume variation that was shared between any pair of social brain regions in our UKBB participants.

### Technical note on neuroscientific interpretation of model coefficients

From a quantitative perspective, compared to other imaging neuroscience work, our study departs in important ways from linear latent factor modeling approaches, such as partial least squares, reduced-rank regression, and canonical correlation analysis. Such dimensionality-reducing tools would necessarily have considered all our 40 traits in conjunction, that is, operating exclusively on the totality of input variables: how a linear combination of the entire set of trait variables explains variation in a region volume at hand. These latent factor models naturally ask for shared variation among the collective trait variables that have a joint relationship with region variation. Instead of aiming at correlated variable effects on a volumetric outcome, our elected multiple regression approach asked for the *marginal* relationship of each individual trait variable with the region volume, in direct competition with the 39 remaining traits examined in our study (i.e., conditioning effects). In doing so, the 40 trait variables could be weighed against each other to dissociate each trait’s separate role in explaining region variation. For example, if a fraction of volume variation in the frontal pole was jointly explained by personality trait A and demographic trait B, then the one of the two corresponding trait parameters with the bigger magnitude of linear association would be attributed the dominating role; since it explains more region volume compared to the competing other trait. Thus, our quantitative approach aimed at singling out each trait’s unique role for tracking interindividual differences in a social brain region, which is uncorrelated from the collection of candidate traits.

### Statistics and reproducibility

To verify if our Bayesian regression analyses generalize to other datasets with the same trait indicators, we have implemented the identical data analysis pipeline (cf. above) in several new, independent participant samples. For this purpose, we used the recently available 40,000 participant release from the UK Biobank (Data Access Application: 25163). To assess replicability, the unseen ~30,000 participants were randomly divided into three new samples of ~10,000 participants each. The regression modeling workflow from the main analysis was carried out again in each of the three data splits. Subsequently, Pearson correlation coefficients were computed between the original analysis and the three new analyses based on the mean of posterior parameter distributions (Supplementary Fig. 6). This re-assessment confirmed the replicability of the constellation of findings obtained in our original analysis in unseen participant samples.

### Reporting summary

Further information on research design is available in the Nature Research Reporting Summary linked to this article.

## Supplementary information

Supplementary Information

Description of Additional Supplementary Files

Supplementary Data 1

Supplementary Data 2

Supplementary Data 3

Reporting Summary

## Data Availability

Source data underlying Figs. 2, 4, and 5, as well as Supplementary Fig. 4–5, are presented in Supplementary Data 3. All used data are available to other investigators online (ukbiobank.ac.uk), or available from the corresponding author upon reasonable request.

## Code availability

All analyses conducted for the present study are reproducible and the scripts used for the analysis pipelines are available online (https://github.com/hannahkiesow/social_brain_aging).
