# Supplementary material for: Dissecting the midlife crisis: disentangling social, personality and demographic determinants in social brain anatomy
Source: Commun Biol. 2021 Jun 17;4:728. doi: 10.1038/s42003-021-02206-x (PMC8211729; doi:10.1038/s42003-021-02206-x)
Supplement: Supplementary file 1 — Supplementary Information [file 42003_2021_2206_MOESM1_ESM.pdf]

Dissecting the midlife crisis: Disentangling social, personality and demographic determinants in social brain anatomy

Hannah Kiesow, Lucina Q. Uddin, Boris C. Bernhardt, Joseph Kable, Danilo Bzdok

Supplementary materials

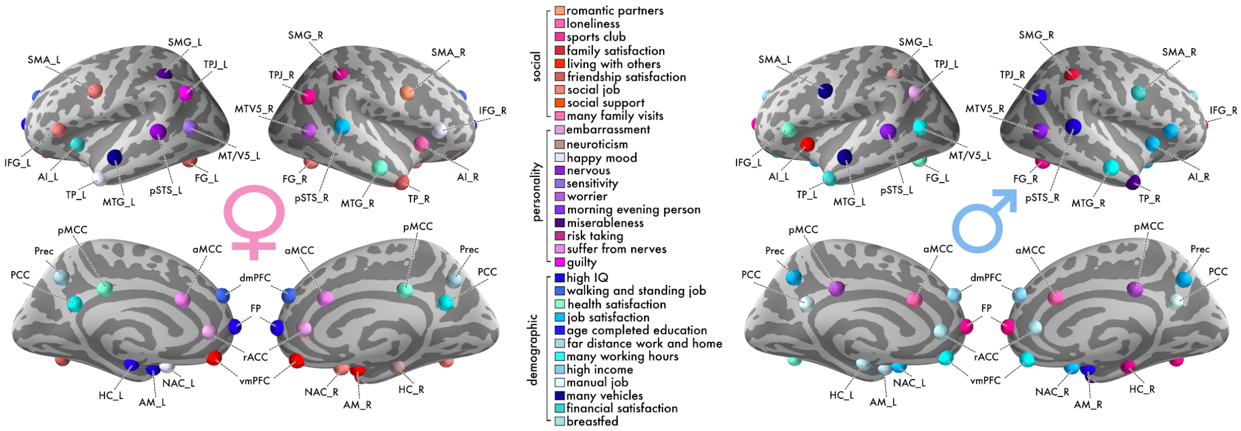

Supplementary Figure 1. **The partial volume analysis reveals a larger variety of dominant trait associations in the social brain.** We applied an additional partial correlation step on each of the 36 social brain regions using a linear de-correlation procedure. This complementary partial correlation analysis goes one step further and accounts for the unique variance of each of the traits in addition to accounting for the companion effects from the other social brain regions before the main probabilistic analysis (cf. Materials and Methods). After the partial correlation step, one generative probabilistic model was applied to each social brain region in our population sample of middle-aged adults. Compared to the main analysis (cf. Fig. 1), these region-by-region analyses revealed a wider variety of dominant trait associations. Colors indicate which individual traits have the largest magnitude (i.e., strongest positive or negative association with region volume) in explaining social brain grey matter region volume, relative to any other of the 39 out of 40 examined candidate traits. Red indicates markers in the social trait category, purple of the personality trait category and blue of the demographic trait category (cf. Table 3 for a description of the social brain region abbreviations). Collectively, the partial volume analysis revealed dominant trait associations that were mostly related to one's occupation. For women, having a job with frequent social interaction was the most common dominant trait association, as seen in the FG, IFG, NAC and SMA (left). For men, feeling satisfied with one's occupation was the most common brain-trait association, as seen in the AI, IFG, NAC and Prec (right).

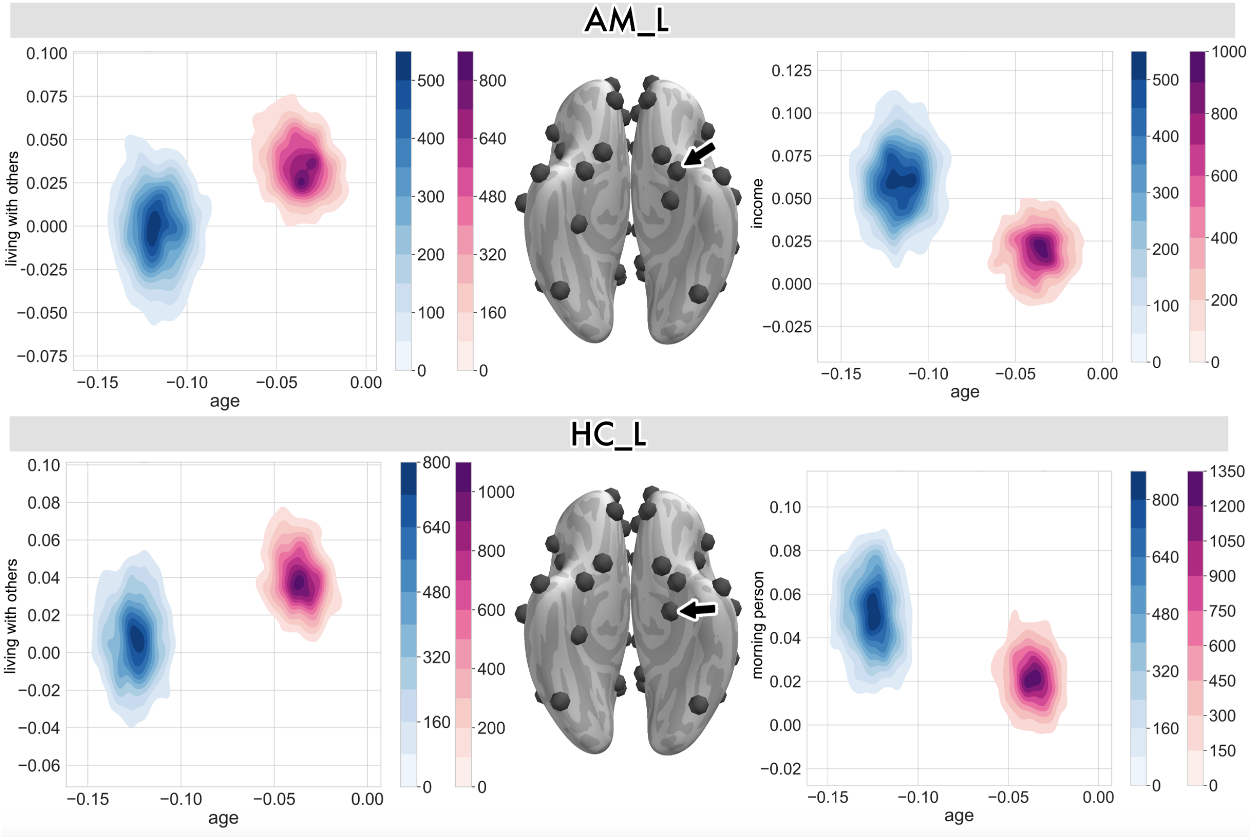

Supplementary Figure 2. Participant age drives how dominant lifestyle traits are linked to midline social brain regions. The co-relationship between age and a trait association is quantified by the joint posterior parameter distribution for one particular social brain region (black arrow) for men (blue) and women (pink). This summary visualization exposes the traits with top effects in the full analysis (cf. Fig. 1). The left column shows these brain-trait associations for women and the right column shows the top trait contributions for men. Middle-aged men and women show diverging age-trait associations in AM_L volume in the context of social interaction frequency and demographic status. The joint posteriors of trait associations in the HC_L show social interaction quality and personality as measured by being a morning person to show non-overlapping posterior distributions between middle-aged men and women (cf. Fig. 3 for midline region results from the main analysis; cf. Table 3 for a description of the social brain region abbreviations). Error bars/dispersion shows uncertainty of Bayesian posterior parameter distributions.

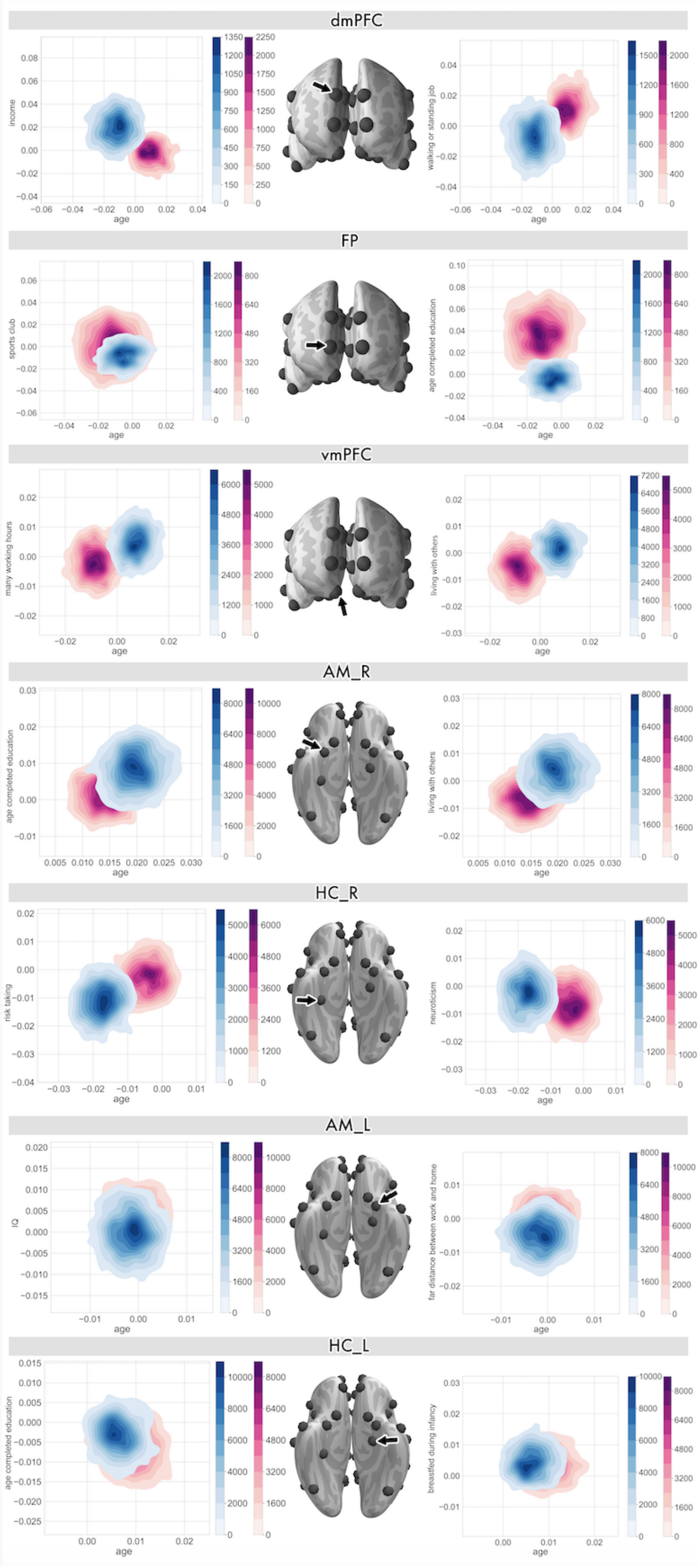
dmPFC
income
age
walking or standing job
FP
sports club
age completed education
vmPFC
many working hours
living with others
AM_R
age completed education
living with others
HC_R
risk taking
neuroticism
AM_L
IQ
far distance between work and home
HC_L
age completed education
breastfed during infancy

Supplementary Figure 3. Participant age drives how dominant lifestyle traits are linked to midline social brain regions. The co-relationship between age and a strong trait association is quantified by the joint posterior parameter distribution for one particular social brain region (black arrow) for men (blue) and women (pink). The left column highlights the top trait associations for women and the right column depicts the dominant traits for men (cf. Supplementary Fig. 1). The depicted results here are from the partial correlation analysis (cf. Fig. 3 for results from the main analysis; cf. Table 3 for a description of the social brain region abbreviations). Error bars/dispersion shows uncertainty of Bayesian posterior parameter distributions.

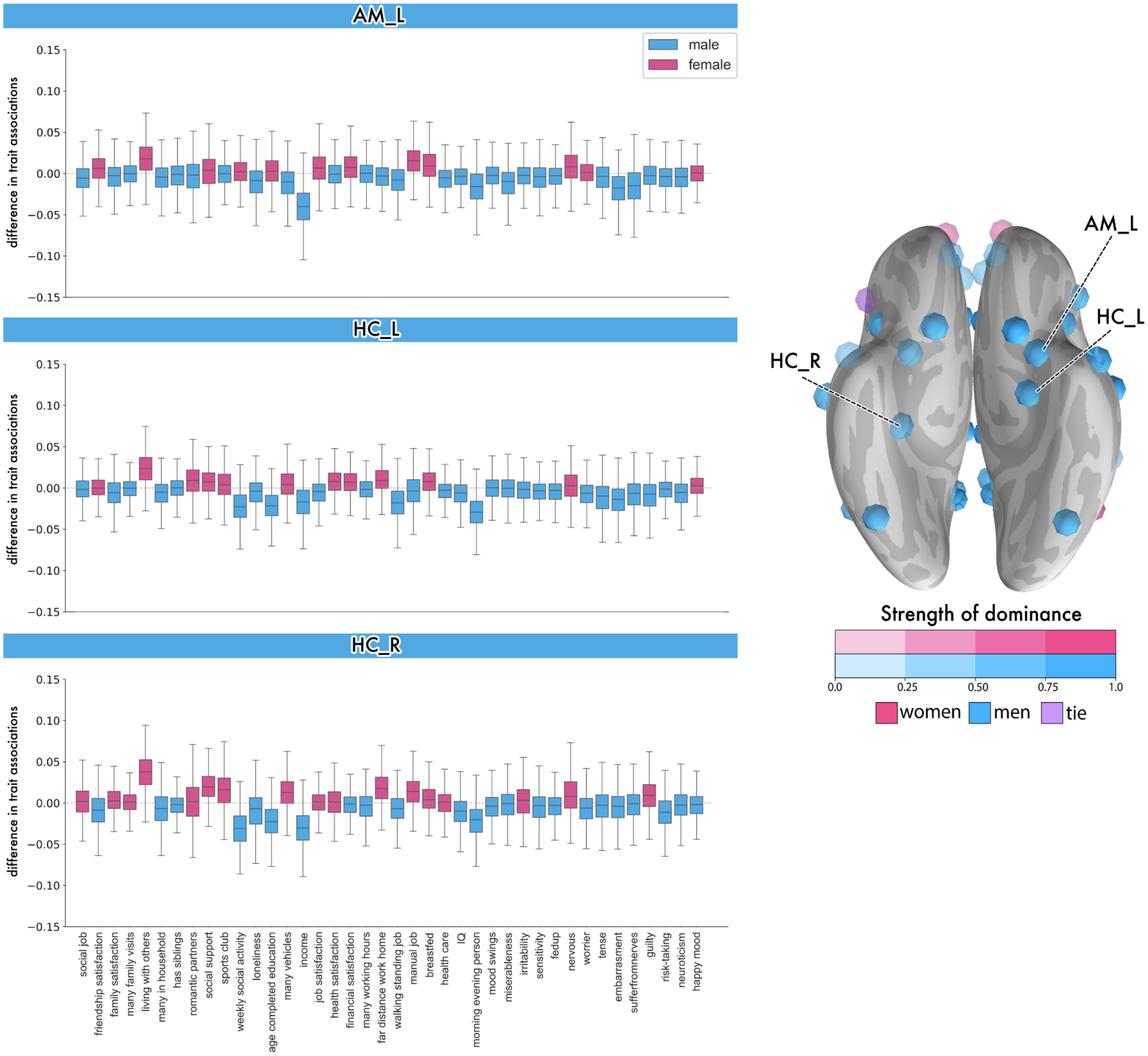

Supplementary Figure 4. Degree of sex bias in brain-trait associations in the social brain midline. Left: In each of the 40 examined traits, boxplots depict the difference contrasts between the marginal posterior population distributions of each sex (female – male). Posterior distribution

means above zero indicate a relatively female-biased effect for that specific trait association (pink). For values below zero, there is a relatively male-biased effect for that specific trait (blue). Right: As a summary visualization, for each brain region, we counted across the 40 trait associations how many were biased predominantly towards males (blue) or more females (pink). Purple shows an equal number of male- and female-biased trait associations. Transparency indicates the strength of the sex divergence. Overall, a male bias in volume effects becomes apparent in the limbic social brain regions. In the AM_L and bilateral hippocampus regions, a predominantly male bias in trait effects becomes apparent. In the AM_L, yearly income showed a stronger male effect for men than women. However, in the bilateral hippocampus, women showed the stronger trait effect than men for the social trait of living with other individuals (cf. Fig. 5 for midline region results from the main analysis; cf. Table 3 for a description of the social brain region abbreviations). Error bars/dispersion shows uncertainty of Bayesian posterior parameter distributions.

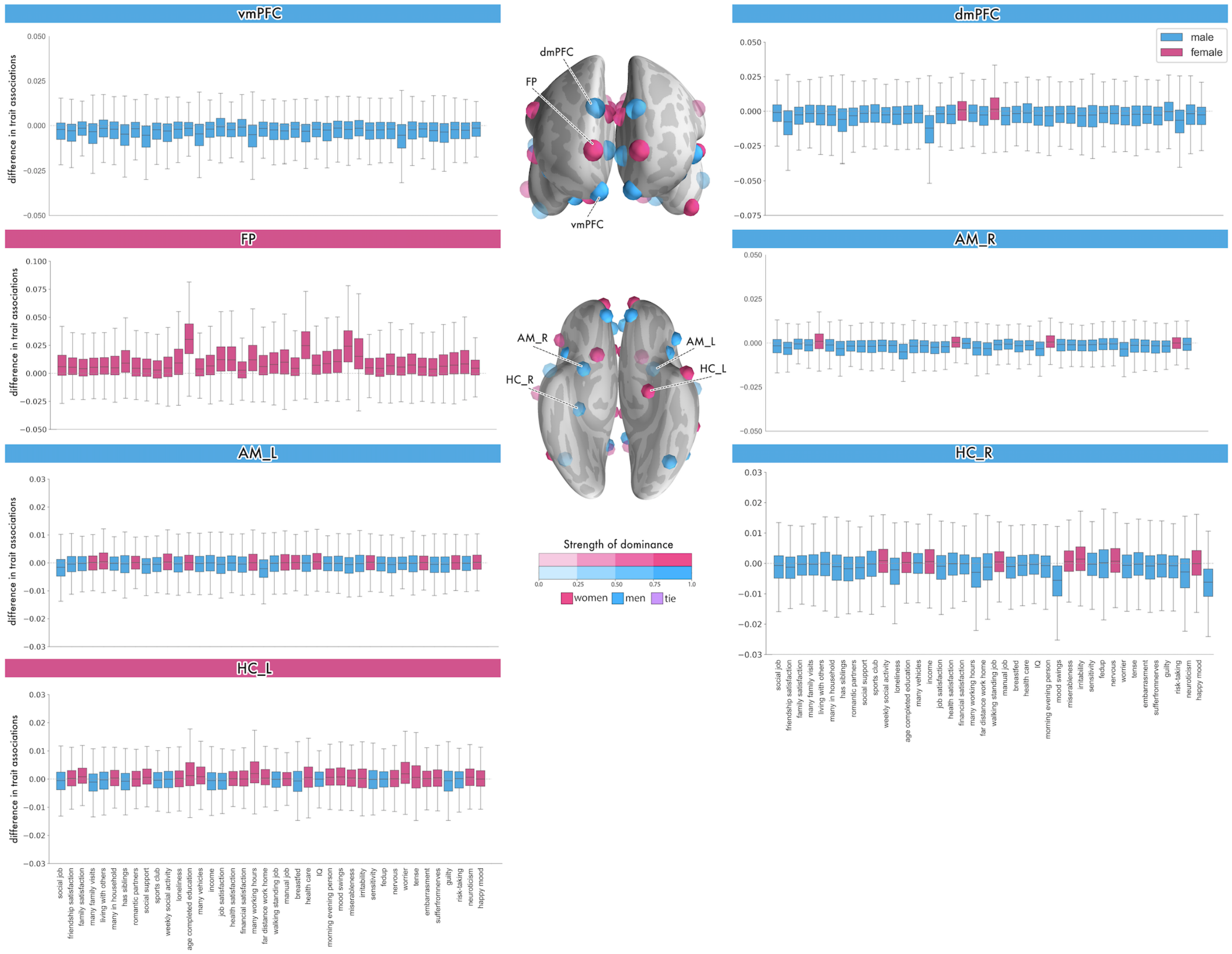

Supplementary Figure 5. Degree of sex differentiation in lifestyle trait associations in social brain midline regions. Left/Right: Boxplots depict the difference contrast of the marginal

posterior population distributions from each sex (female – male) in each of the 40 examined lifestyle traits for the social brain midline. As such, distribution peaks above zero indicated a female-biased effect for that specific trait association (pink). For values below zero, there is a male-biased effect for that specific trait (blue). Middle: As a summary visualization, for each brain node, we counted across the 40 trait associations how many were biased predominantly towards males (blue) or more females (pink). Purple shows an equal number of male- and female-biased trait associations. Transparency indicates the strength of the sex effect. The depicted results here are from the partial correlation analysis (cf. Fig. 5 for results from the main analysis; cf. Table 3 for a description of the social brain region abbreviations). Error bars/dispersion shows uncertainty of Bayesian posterior parameter distributions.

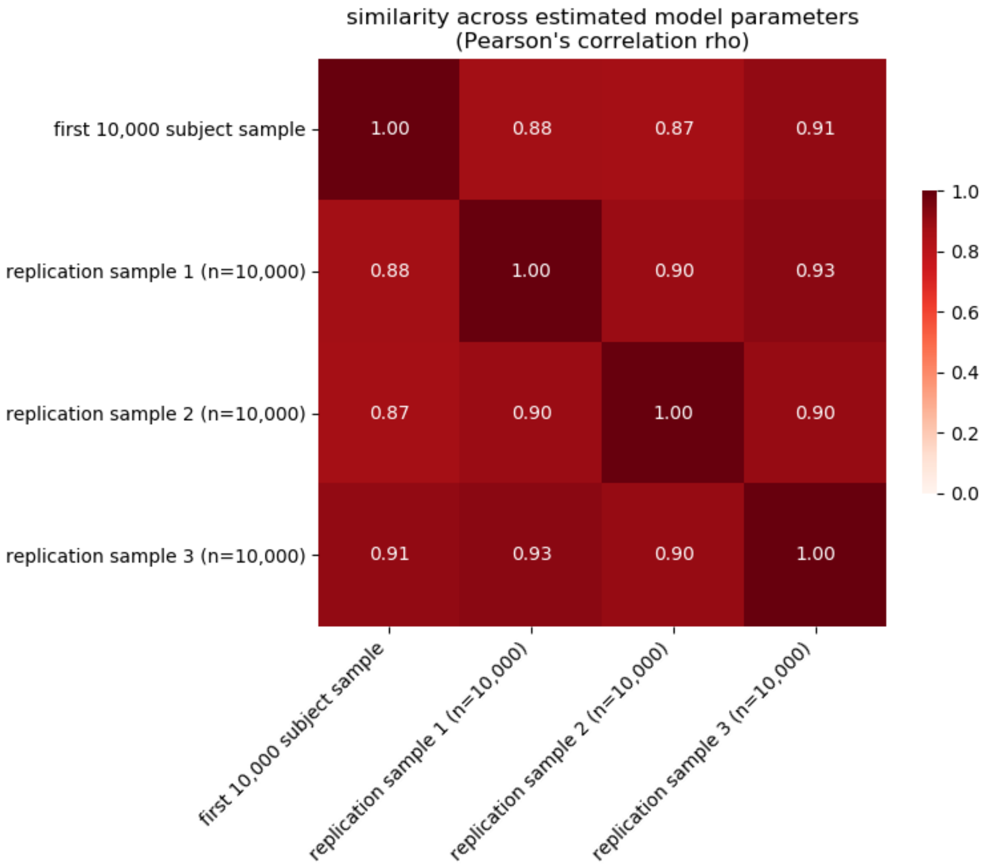

Supplementary Figure 6. The regression modeling results replicate in independent data. The regression estimates from the main analysis (methods section) were repeated in three samples of ~10,000 unseen participations from the recent 40,000 participant UK Biobank release. Pearson's correlation coefficient (best possible outcome: 1.00) was computed across collective model

parameters from the original and the three new participant samples. The three repetitions of our analysis corroborated our results and conclusion from the original 10,000 participant sample.

Supplementary Note: Partial volume analysis

In comparison to the main probabilistic regression analysis, the partial correlation analysis revealed a wider variety of unique brain-trait associations in all three trait domains for our middle-aged participants.

At the level of interpersonal exchange, the partial volume analysis results further revealed additional variety in the extent to which qualitative aspects of social networks contribute to region volume during the midlife milestone (Supplementary Fig. 1). For example, sharing the home environment with other individuals showed dominant trait effects in several limbic and intermediate network regions including the AM and vmPFC (women: AM_R: mean of the population trait posterior distribution = -0.006, highest density interval of the population trait posterior distribution covering 95% uncertainty (HPDI) = -0.017 – 0.004; vmPFC: posterior mean = -0.005, HPDI = -0.017 – 0.006; men: AI_L: posterior mean = -0.016, HPDI = -0.040 – 0.006). Moreover, the quality of social engagement with close family members and friends showed several dominant trait effects, and were most evident in regions of the intermediate network. For example, feeling satisfaction with family members showed a trait effect in the SMG (men: SMG_R: posterior mean = -0.010, HPDI = -0.036 – 0.010), as well as the amount of family visits in the aMCC (men: aMCC: posterior mean = -0.007, HPDI = -0.023 – 0.005). Similarly, growing up with brothers and sisters, as opposed to being an only child, showed a dominant trait effect in the CB_L (women: CB_L: posterior mean = 0.006, HPDI = -0.004 – 0.017).

In the context of close interpersonal relationships, traits indexing close, emotional bonds showed trait effects in several social brain regions of the visual sensory, limbic, intermediate and higher associative networks. In particular, the lifetime number of romantic partners contributed most to region volume in the intermediate SMA_R region (women: SMA_R: posterior mean = -0.017, 95% HPDI = -0.044 – 0.007). Similarly, feeling happy with one's friendships (women: FG_L: posterior mean = -0.008, HPDI = -0.024 – 0.006; TP_R: posterior mean = 0.006, HPDI = -0.007 – 0.019) and higher amounts of social support from close others showed several dominant trait effects (men: CB_R: posterior mean = -0.008, HPDI = -0.023 – 0.006). Correspondingly, feelings of loneliness showed a strong trait effect in the AI (women: AI_R: posterior mean = -0.004, HPDI = -0.015 – 0.006). Sports club membership, an indicator of social exchange in extracurricular activities, explained the most region volume in several higher associative and visual sensory social brain regions including the TPJ and FP (men: FG_R: posterior mean = 0.015, HPDI = -0.003 – 0.036; FP: posterior mean = -0.008, HPDI = -0.029 – 0.010; women: TPJ_R: posterior mean = -0.022, HPDI = -0.053 – 0.001). In sum, compared to the original volume analysis, the

complementary partial volume analysis revealed a wider scope of traits describing the quality of social bonds to contribute to region volume. In particular, social aspects of one's occupation were observed to contribute most to region volume.

Focusing on trait effects in the personality category, our partial region-by-region volume analysis revealed a wider variety of personality traits to explain social brain region volume in our middle-aged population sample (Supplementary Fig. 1). We found the personality trait of being a morning, as opposed to evening person, to explain the most variation in several regions of the visual sensory network (men: MTV5_L: posterior mean = 0.000, 95% HPDI = -0.021 – 0.021; pSTS_L: posterior mean = -0.003, HPDI = -0.010 – 0.003)). Additionally, personality traits linked to well-being explained the largest fraction of volume variation in several social brain regions compared with the other considered traits. For example, having a happy mood showed the largest dominant trait effect in several limbic, intermediate and higher associative network regions including the IFG (women: IFG_R: posterior mean = 0.016, HPDI = -0.005 – 0.043), NAC (women: NAC_L: posterior mean = 0.028, HPDI = 0.004 – 0.055) and TP (women: TP_L: posterior mean = -0.006, HPDI = -0.023 – 0.007). Moreover, neuroticism, a personality trait, showed dominant trait associations in several limbic and intermediate network regions (women: HC_R: posterior mean = -0.007, HPDI = -0.019 – 0.006; men: SMG_L: posterior mean = -0.011, HPDI = -0.036 – 0.010).

Additional traits indexing neurotic behavior were found to show the largest magnitude in explaining social brain volume in several limbic and higher associative regions compared to the other examined traits. These personality traits include feelings of embarrassment (women: rACC: posterior mean = 0.008, 95% HPDI = -0.008 – 0.027; men: TPJ_L: posterior mean = -0.010, HPDI = -0.028 – 0.007), feelings of miserableness (women: SMG_L: posterior mean = 0.014, HPDI = -0.010 – 0.041; men: TP_R: posterior mean = 0.006, HPDI = -0.007 – 0.020). Similarly, additional personality traits related to neuroticism were also found to be uniquely associated with region volume in regions of the visual sensory, intermediate and higher associative networks such as being a 'worrier' (women: MTV5_R: posterior mean = -0.018, HPDI = -0.045 – 0.005; men: pMCC: posterior mean = -0.017, HPDI = -0.040 – 0.003), feelings of guilt (women: CB_R: posterior mean = 0.004, HPDI = -0.006 – 0.015; TPJ_L: posterior mean = -0.015, HPDI = -0.041 – 0.009), nervous feelings (women: pSTS_L: posterior mean = -0.009, HPDI = -0.032 – 0.011), feelings of sensitivity (women: MTV5_L: posterior mean = 0.008, HPDI = -0.009 – 0.032), and suffering from nerves (women: aMCC: posterior mean = 0.010, HPDI = -0.008 – 0.036).

In addition to personality trait effects related to well-being, participants who identified themselves as risk-takers showed dominant trait associations in the SMG (women: SMG_R: posterior mean = 0.008, 95% HPDI = -0.011 – 0.029) and HC (men: HC_R: posterior mean = -0.011, HPDI = -0.024 – 0.000). Taken together, our partial volume analysis revealed personality traits related to well-being to show the most frequent dominant trait associations in a number of social brain regions.

Regarding experiences at the broader societal level, the partial volume analysis revealed a more fine-grained profile of demographic trait effects during midlife. Specifically, traits related to

one's occupation showed dominant brain-trait associations in several social brain regions. In particular, feelings of satisfaction with one's job showed strong contributions to explaining region volume in mostly visual sensory, limbic and intermediate regions, including the bilateral NAC, AI and IFG (men: NAC_L: posterior mean = -0.025, 95% HPDI: -0.052 – 0.000; NAC_R: posterior mean = 0.007, HPDI = -0.006 – 0.021; AI_R: posterior mean = -0.011, HPDI = -0.032 – 0.002; IFG_R: posterior mean = 0.010, HPDI = -0.011 – 0.034; women: pSTS_R: posterior mean = 0.010, HPDI = -0.004 – 0.030).

In addition, working more than 40 hours a week explained the largest fraction of volume variation in several visual sensory, limbic and higher association regions including the vmPFC (men: MT/V5_L: posterior mean = -0.010, 95% HPDI = -0.032 – 0.009; vmPFC: posterior mean = 0.005, HPDI = -0.006 – 0.016; MTG_R: posterior mean = 0.007, HPDI = -0.008 – 0.024; women: PCC: posterior mean = 0.012, HPDI = -0.004 – 0.030). Furthermore, demographic aspects related to the complexity of one's occupation showed the largest population trait effects in several higher associative social brain regions, compared with the other considered traits. For example, having a far distance between work and home showed a dominant trait effect in the AM And Prec (men: AM_L: posterior mean = -0.005, HPDI = -0.016 – 0.004; women: Prec: posterior mean = 0.009, HPDI = -0.007 – 0.030). Similarly, having a job that requires walking or standing for most of the workday showed a trait effect in the higher associative dmPFC (women: dmPFC: posterior mean = 0.012, HPDI = -0.006 – 0.035), whereas working a manual job contributed most to region volume in the PCC (men: PCC: posterior mean = 0.012, HPDI = -0.004 – 0.031).

Intimately linked to aspects of one's occupation, several demographic markers related to income explained the largest fraction of volume variation in social brain regions of the intermediate and higher associative networks. For example, satisfaction with one's financial status contributed most to explaining social brain volume in several intermediate and higher associative network regions including the AI and SMA (male: SMA_R: posterior mean = 0.004, 95% HPDI = -0.008 – 0.018; TP_L: posterior mean = 0.011, HPDI = -0.004 – 0.030; women: AI_L: posterior mean = -0.010, HPDI = -0.027 – 0.006). In an index of affluence, we observed that the number of vehicles owned showed strong trait effects (men: MTG_L: posterior mean = -0.014, HPDI = -0.038 – 0.007; SMA_L: posterior mean = 0.010, HPDI = -0.008 – 0.030; women: MTG_L: posterior mean = -0.011, HPDI = -0.034 – 0.010). Furthermore, job income explained the largest fraction of volume variation in the dmPFC (men: dmPFC: posterior mean = 0.021, HPDI = -0.006 – 0.050).

In aspects related to education, several demographic markers revealed large magnitudes in explaining grey matter volume in several social brain regions. For example, age of full education attainment showed the largest magnitude in explaining region volume in several higher order and limbic areas including the FP, AM and TPJ (men: AM_R: posterior mean = 0.009, 95% HPDI = -0.002 – 0.020; TPJ_R: posterior mean = 0.009, HPDI = -0.008 – 0.030; women: FP: posterior mean = 0.039, HPDI = 0.008 – 0.075; HC_L: posterior mean = -0.006, HPDI = -0.016 – 0.003). Moreover, having a high IQ showed a dominant trait effect in the AM (women: AM_L: posterior mean = 0.004, HPDI = -0.004 – 0.012) and pSTS_R (men: posterior mean = -0.008, HPDI = -0.028 – 0.010). In addition, we observed that satisfaction with one's health explained region

volume in several social brain areas (men: FG_L: posterior mean = -0.015, HPDI = -0.042 – 0.005; IFG_L: posterior mean = 0.016, HPDI = -0.006 – 0.042; women: MTG_R: posterior mean = -0.012, HPDI = -0.031 – 0.004; pMCC: posterior mean = -0.006, HPDI = -0.018 – 0.005). Taken together, the dominant trait associations in the demographic category show that during midlife, social status and aspects of one's occupation were top contributors of explaining social brain grey matter volume.
