# Supplementary material for: Dissecting the midlife crisis: disentangling social, personality and demographic determinants in social brain anatomy
Source: Commun Biol. 2021 Jun 17;4:728. doi: 10.1038/s42003-021-02206-x (PMC8211729; doi:10.1038/s42003-021-02206-x)
Supplement: Supplementary file 2 — Description of Additional Supplementary Files [file 42003_2021_2206_MOESM2_ESM.pdf]

Description of Additional Supplementary Files

File name: Supplementary Data 1

Description: List of the 40 examined traits available in the UK Biobank. Each lifestyle indicator of interest from UK Biobank participants is shown alongside its field identification number. Each indicator was analyzed in two groups according to sex. All indicators were divided into one of three categories, defined by traits related to regular social interaction (social domain), traits related to personality (personality domain) and traits related to demographic standing and environment (demographic domain).

File name: Supplementary Data 2

Description: Social brain atlas regions and their MNI coordinates. Social brain regions and their respective functional network.

File name: Supplementary Data 3

Description: Source data underlying figures.
